# Modeling Rare Events and Nonmonotone Nonignorable Missingness of Time-Varying Outcomes and Predictors in Binary Time-Series Daily Diary Data: A Bayesian Selection Model

**DOI:** 10.1017/psy.2026.10120

**Published:** 2026-06-08

**Authors:** Sun-Joo Cho, Autumn Kujawa, Corinne Carlton, Yinru Long, Rachel Marlowe

**Affiliations:** Vanderbilt University, USA

**Keywords:** Bayesian analysis, daily diary data, mixed-effects complementary log–log regression, nonmonotone nonignorable missingness, selection model

## Abstract

This study investigates the relationship between daily interpersonal stress (binary, time-varying) and suicidal behavior (binary, time-varying) using 90 days of daily diary data from 106 adolescents assessed immediately after discharge from acute psychiatric treatment. It addresses two key complexities: the rarity of suicidal events and non-monotone, non-ignorable missingness in both the outcome and the predictor. Because existing methods often fail to accommodate these complexities, leading to biased estimates, a Bayesian selection model is specified. The model integrates a mixed-effects complementary log–log regression for rare events with a missingness model that accounts for non-monotone, non-ignorable missingness in the outcome. A probit mixed-effects model is used for the time-varying predictor, along with a corresponding missingness model for its non-monotone, non-ignorable missingness. Empirical results support the applicability of the specified model to longitudinal studies involving rare events and complex missing-data structures. Furthermore, a simulation study demonstrates parameter recovery and highlights bias in focal parameters when sensitivity parameters in the outcome and missingness models are ignored.

## 1 Introduction

### 1.1 Empirical motivation

This study addresses challenges in investigating the relationship between daily interpersonal stress (a binary, time-varying predictor) and suicidal behavior (a binary, time-varying outcome) using 90 days of daily diary data from 106 adolescent participants assessed immediately following discharge from acute psychiatric treatment, while also exploring the associations between baseline variables and suicidal behaviors. In this study, suicidal behavior is defined as actions taken with the intent to die and includes actual, interrupted, and aborted suicide attempts, assessed via daily self-report. In the motivating dataset, suicidal behavior is a rare event, with only 22 instances of endorsement (0.23%) from 17 participants, compared to 5,821 instances of non-endorsement (61.02%) and 3,697 instances of missing data (38.75%). This extreme sparsity makes it statistically challenging to model and analyze. In addition, the substantial proportion of missingness in binary time-varying outcomes and predictors further complicates the analysis. Among the 9,540 total observations, 1,336 (14.00%) indicated endorsement of the daily interpersonal stress variable, 4,506 (47.24%) indicated nonendorsement, and 3,698 (38.76%) were missing. To better understand and address this issue, our research team coded potential reasons for the missingness. The majority of these reasons were classified as nonignorable missingness, where the likelihood of missing data is related to the unobserved values themselves, and the missingness does not follow a straightforward pattern. This characteristic poses unique challenges for statistical analysis, necessitating specialized methods to ensure accurate parameter estimation of the effects of interest (e.g., the relationship between daily interpersonal stress and suicidal behavior). Accordingly, this study aims to specify a model capable of examining the relationship between daily interpersonal stress and suicidal behavior, while accounting for data complexities.

### 1.2 Data complexities and limitations of current approaches

The daily diary data in our motivating study involve complexities, such as rare events and nonmonotone nonignorable missingness, each of which presents unique challenges for analytical approaches. The following discuss the limitations of existing methods for addressing each of these challenges.

#### 1.2.1 Modeling rare events in binary time-series data

Suicidal behaviors present distinctive modeling challenges because they are infrequent yet consequential. Standard binary-response links based on symmetric latent-error assumptions (e.g., standard normal or logistic distributions) may be poorly aligned with the asymmetric behavior expected when event frequencies are very low. By contrast, extreme-value models—most notably the Gumbel distribution—are suited to characterizing tail behavior and rare occurrences (Fisher, 1922), and are widely used in domains where extreme outcomes carry substantial implications (e.g., meteorology and finance; Coles, 2001). The Gumbel distribution has also appeared in psychometric applications (e.g., Goldstein, 1980; Shim et al., 2023), but, to our knowledge, has not been applied to binary modeling of clinical outcomes such as suicidal behaviors. It is also well recognized that, for rare outcomes, predicted probabilities from the logit link (based on the logistic distribution) and the complementary log–log (cloglog) link (based on the Gumbel distribution) are often very similar. Differences in predicted probabilities are typically small when probabilities are below 0.10 and remain modest between 0.10 and 0.20 (Austin & Fine, 2017). Even so, choosing a Gumbel-based specification is theoretically justified in this study because it accommodates the expected asymmetry for suicidal behaviors and supports a hazard-oriented interpretation of effects, features that align with the questions motivating this study.

#### 1.2.2 Modeling nonmonotone nonignorable missingness

In our motivating empirical study, we coded a primary reason for missingness for most participants to explain why they did not respond. Some reasons could potentially lead to a *missing not at random* (MNAR; Rubin, 1976) missing data mechanism. For example, participants may have opted out of surveys because of high levels of stress and/or because they did not want to disclose recent suicidal thoughts and behaviors, which influences both their survey completion behavior and potentially other unobserved responses, creating a dependency between the missingness and the unobserved data. In addition, participants who engage in suicidal behavior may need to return to acute psychiatric treatment which could prevent survey completion, and missingness may correlate with values that would have been observed had the data been complete. Furthermore, *non-monotone missing data* were observed. They indicates that participants had missing data at one point in time, provided data at another point, and then have missing data again at a later point, resulting in gaps that are not consistently cumulative.

Commonly used missing data treatments (see Ibrahim & Molenberghs, 2009, for a review) have been applied to intensive longitudinal and time-series data. Cursio et al. (2019) applied a shared parameter model (Wu & Carroll, 1988) to intensive longitudinal data, in which the shared parameter represents between-person differences in response willingness. McNeish (2025) employed the Diggle–Kenward selection model (Diggle & Kenward, 1994) to account for MNAR intensive longitudinal binary outcomes within a dynamic structural equation model (Asparouhov et al., 2018). In the current study, a selection model (Diggle & Kenward, 1994; Heckman, 1976; Little & Rubin, 2020) was chosen to address MNAR in time-varying outcomes and predictors. The selection model is more appropriate than the shared parameter model because it enables the modeling of missingness in an outcome variable as a function of the outcome itself at the individual level, rather than between-person differences, as in the shared parameter model. As McNeish (2025) also pointed out, the selection model is better suited to handling nonmonotone missing data compared to the pattern-mixture model (Little, 1983), which requires modeling the outcome separately for each missing-data pattern and then combining these models based on their distribution. This approach poses a significant implementation challenge given the large number of time points (90 days in our study) and the presence of intermittent missingness (Lin et al., 2018), which result in a substantial number of missing-data patterns. Another reason for preferring the selection model over the pattern-mixture model is that the primary focus of inference in the current study is on the conditional distribution of the outcome, whereas pattern-mixture models primarily target marginal distributions within patterns, requiring additional assumptions to recover conditional relationships (e.g., Ibrahim & Molenberghs, 2009). However, to our knowledge, the selection model has not yet been applied to rare events in binary time-series data with MNAR in time-varying outcomes and predictors.

### 1.3 Study purpose

To address the limitations outlined previously, the aim of the current study is to specify a selection model comprising: (a) a mixed-effects cloglog regression for rare events, derived from the Gumbel-based latent variable construction (serving as the substantive model or full-data response model); (b) a missingness model for MNAR time-varying outcomes; (c) a probit mixed-effects model as the substantive model for an MNAR time-varying predictor; and (d) a missingness model for an MNAR time-varying predictor, in binary time-series daily diary data. A Bayesian approach is chosen for parameter estimation due to its flexibility in handling complex models using the WinBUGS (Spiegelhalter et al., 2007). This approach allows simultaneous implementation of model parameters, imputation of missing data, and assessment of model adequacy within a unified probabilistic framework. The specified model is applied to a motivating dataset to test the hypothesis that daily interpersonal stress predicts suicidal behavior. In addition, a simulation study is conducted to evaluate parameter recovery for the specified model and to demonstrate the consequences of ignoring *sensitivity parameters*. These parameters quantify departures from the MAR assumption by capturing the dependence of the missingness mechanism on the unobserved values of partially observed outcomes or predictors, and are required to identify and estimate focal parameters under MNAR. These aspects have not been previously demonstrated in the literature.

This article is organized as follows. In Section 2, we describe the empirical study that motivated the current work. Section 3 presents the specified model, including parameter estimation, missing data imputation, and model adequacy checking. In Section 4, we illustrate the specified model using an empirical data set. Section 5 presents a simulation study to evaluate the model. Finally, Section 6 concludes with a summary and discussion.

## 2 Motivating empirical study

In this section, the motivating empirical study is explained.

### 2.1 Empirical study design and measures

Adolescent suicidal thoughts and behaviors are a major clinical concern, but there are few established prospective predictors of suicidal thoughts and behaviors, much less an understanding of when suicidal thoughts and behaviors are likely to occur. Furthermore, much of the literature focuses on predicting suicidal ideation due in part to relatively low frequencies of suicidal behaviors, particularly in general populations and at specific time points. The months following acute psychiatric treatment are a high-risk time for suicidal behaviors, and leveraging intensive longitudinal designs with questionnaires completed on mobile phones (e.g., daily survey and ecological momentary assessment [EMA]) can provide important insights into factors that immediately precede suicidal behaviors during this high-risk period. The present study examines the relationship between daily interpersonal stress and suicidal behaviors using daily diary data, and further explores associations with baseline variables.

Participants were recruited through the partial hospitalization and inpatient treatment programs at a large university medical center in the southeastern United States. Inclusion criteria were adolescents between the ages of 13 and 17; actively treated through partial hospitalization or inpatient treatment at the medical center with recent non-suicidal self-injury (NSSI), suicidal ideation, and/or suicidal behaviors; and adolescent and caregiver able to read and speak fluently in English. Exclusion criteria included a current active psychotic or manic episode. Additionally, the presence of cognitive impairments, intellectual disabilities, or vision/hearing deficits that could interfere with the ability to complete measures was exclusionary. The analyses included 106 adolescents, and their demographic information is summarized in Appendix A.

Beginning on the day of hospital discharge, adolescents completed a brief daily diary survey in the evening (5–8 pm) for 90 consecutive days. To reduce burden and standardize responses, most items were administered as yes/no questions, yielding a binary daily indicator of suicidal behavior. Each day, participants reported whether, since the previous survey, they had engaged in a suicide attempt, had an aborted attempt, or had an interrupted attempt. Endorsements triggered a follow-up safety assessment by clinically trained staff; these assessments were conducted via direct phone contact to verify the nature and timing of the reported behavior. When reports were judged to reflect misunderstanding (e.g., increased suicidal ideation rather than behavior), responses were recoded accordingly. This verification process was used to distinguish true suicidal behaviors from reporting errors or misinterpretations. For analysis, suicidal behavior was collapsed across the three attempt categories and coded as present ( =1 ) if any category was endorsed and absent ( =0 ) otherwise. This design yields a 90-day series of binary suicidal behavior observations per participant as the time-varying outcome variable.

Participants completed a *daily* checklist of interpersonal stressors prompted by “Check all that happened to you since the last survey you completed,” with 12 options: problem or argument with friend/another teen, parent, family member, significant other/dating partner, or someone else; bullied/teased; discrimination based on sexual orientation, gender identity, race, or ethnicity; ignored/rejected; breakup with significant other/dating partner; end of a friendship; other stressful situation/event; or none of these. Because the survey assessed experiences on a daily basis, multiple stressors within a single day were not expected to be common; accordingly, emphasis was placed on distinguishing between days with versus without any interpersonal stressor. Consistent with this, 14% of surveys endorsed any interpersonal or “other” stressful event, and only 5% included more than one event. In the current study, stressors were collapsed across items for each participant on each day (yielding a time-varying variable) and treated as a binary indicator, coded as endorsed (=1) when at least one of the first 11 options was checked and as not endorsed (=0) otherwise. Although item-specific associations may be of substantive interest, endorsement rates for individual stressors were low (e.g., problems or arguments with parent: 5.6%; discrimination based on sexual orientation: 0.65%), limiting the feasibility of disaggregated analyses.

As baseline clinical and self-report measures, participants completed a set of standardized clinical interviews and self-report questionnaires assessing psychological, behavioral, and contextual correlates of suicidal behavior at baseline. The self-injurious thoughts and behaviors interview–revised (SITBI-R; Fox et al., 2020) was administered by trained Master’s- or Doctoral-level researchers to assess lifetime suicidal and NSSI; lifetime suicide attempt count and history of NSSI were coded as binary indicators (endorsed vs. not endorsed). Trauma history was measured using the Trauma Module of the Schedule for Affective Disorders and Schizophrenia for School-Age Children (K-SADS; Kaufman et al., 1997), yielding a summed score of traumatic event types (0–8). Self-report measures included the Suicidal Ideation Questionnaire-Junior (SIQ-Jr; Reynolds and Mazza, 1999) for current suicidal ideation, the Revised Peer Experiences Questionnaire (RPEQ; Prinstein et al., 2001) for relational, reputational, and overt peer victimization, and the Child and Adolescent Social Support Scale (CASSS; Malecki et al., 1999) for parental and peer/friend support. Depressive and anxiety symptoms were assessed using the short form of the Revised Children’s Anxiety and Depression Scale (RCADS; Ebesutani et al., 2012), and coping flexibility was measured via the Self-Perceived Flexible Coping with Stress Scale (SFCS; Zimmer-Gembeck et al., 2018), which yields subscales for multiple coping strategies, situational coping, and coping rigidity. Finally, sleep disturbance and impairment were assessed using the Patient-Reported Outcomes Measurement Information System (PROMIS) Short Form Sleep Disturbance and Sleep-Related Impairment scales (Forrest et al., 2018). Together, these baseline measures provided a set of time-invariant predictors representing individual differences in psychological functioning, coping, and psychosocial context.

In addition, the study-characteristic time-varying variables comprised survey day (a continuous index of diary day) and two calendar-based indicators: weekend and summer. Survey day captured temporal position within the study period; weekend indicated whether an observation occurred on Saturday or Sunday (1 = yes, 0 = no); and summer indicated whether an observation occurred in July or August (1 = yes, 0 = no). These variables were included as temporal controls to account for secular trends and calendar-related fluctuations that may systematically influence daily outcomes. Appendix A presents the descriptive statistics of all variables considered in the current study.

### 2.2 Data description: Rare event and missingness

The outcome variable, suicidal behavior (time varying), exhibits severe imbalance. In the analytic sample, the positive outcome (“Yes”) is an exceptionally rare event, constituting only 22 cases out of 9,540 total observations ( 0.23% ).

Appendix B displays missingness and endorsement patterns across 90 days for suicidal behavior (top panel) and prior stressor exposure (bottom panel) for each participant (each row). Missing data (red) are prevalent in both panels but appear more frequent and persistent in suicidal behavior, particularly during the latter portion of the diary period. Some individuals show long blocks of missing responses, due in part to study dropout and changes in treatment status. In addition, while most participants did not endorse suicidal behavior (gray), there are isolated instances of endorsement (blue), typically sparse and not clustered. Endorsements of the daily interpersonal stress variable (blue in the bottom panel) are more frequent and distributed across a broader range of days, though many participants also exhibit low endorsement rates. Overall, the patterns suggest non-monotonic missingness and participant heterogeneity in both response and missingness mechanisms.

Reasons for missing daily surveys were evaluated in two ways. First, when sending reminders and communicating with families, staff were occasionally informed of issues related to daily survey completion. These reports were documented and coded as reasons that accounted for missing survey days for each participant. Among the 106 participants included in this article, 9 reported at some point during the protocol that they missed surveys due to technical issues (e.g., messaging system problems), 2 reported being too busy with school or work, 13 were rehospitalized or admitted to residential treatment centers, and 14 withdrew early from the study. In addition, six participants reported other reasons for missing surveys, such as loss of phone access due to legal issues or other circumstances. Next, a subset of participants completed a brief weekly survey (administered at the end of each week for 12 weeks) to assess reasons for not completing all daily questionnaires in that week. This survey was introduced after the study began and was sent only to participants who did not complete all surveys in a given week. Thus, only 64 participants were included. Among them, 329 weekly surveys were sent, of which 132 surveys were completed (40% completion rate). Across all completed weekly surveys on reasons for missing surveys, the most frequently endorsed reason was forgetting to complete the survey that week (52.3%). Other commonly endorsed reasons included being busy with other activities (19.2%), spending time with friends or family (18.9%), and school or work (15.2%). Based on our missing data coding, some missing values in the suicidal behavior and the daily interpersonal stress variable may both reflect an MNAR mechanism. For some participants, missing responses may be influenced both their reported stress levels and their unreported suicidal behavior. For example, participants who report low levels of stress may be less likely to answer the survey or the suicidal behavior item because they feel disengaged from the study or do not perceive the question as relevant to them. Conversely, participants experiencing high levels of stress may miss the survey or the suicidal behavior question if they are in acute distress, actively engaging in suicidal behavior, or in a higher level of care. In this case, both stress and suicidal behavior could contribute to missingness.

## 3 Methods

In this section, a selection model is specified. Subsequently, the Bayesian estimation and model adequacy checks are described.

### 3.1 Model specification

A joint model is often used to correct for non-ignorable missingness (Molenberghs et al., 2014). The selection model (Diggle & Kenward, 1994; Heckman, 1976; Little & Rubin, 2020) is based on the factorization of the joint density of the observed data and the missing data indicators. The selection model has been applied to MNAR outcomes (e.g., Molenberghs et al., 2014), MNAR predictors (e.g., Ibrahim et al., 1999), and MNAR outcomes and predictors (e.g., Du et al., 2022 for a linear regression model and Jackson et al., 2010 for a logistic regression model).

Let $y_{tj}$ be the *t*th observation of a binary outcome (suicidal behavior) of the *j*th participant ( j=1,…,J ; $t=1,\ldots ,T_{j}$ , where $T_{j}=T=90$ in our motivating example). To deal with missingness, the participant-specific outcomes are in a vector $y_{j}=[y_{1j},\ldots ,y_{Tj}{]}^{\prime }$ with an observed component ( $y_{j}^{o}$ ) and a missing component ( $y_{j}^{m}$ ). In addition, for each observation *t*, the missing data indicator $r_{y.tj}$ is defined as $r_{y.tj}=1$ if $y_{tj}$ is observed and $r_{y.tj}=0$ otherwise. The $r_{y.tj}$ values are then grouped into a vector of missing data indicators for each participant, $r_{y.j}=[r_{y.1j},\ldots ,r_{y.Tj}{]}^{\prime }$ . Similarly, let $x_{tj}$ be the *t*th observation of a time-varying predictor for participant *j*, and define the participant-specific predictor vector as $x_{j}=[x_{1j},\ldots ,x_{Tj}{]}^{\prime }$ , with an observed component ( $x_{j}^{o}$ ) and a missing component ( $x_{j}^{m}$ ). The missing data indicator for the predictor, $r_{x.tj}$ , is defined as $r_{x.tj}=1$ if $x_{tj}$ is observed and $r_{x.tj}=0$ otherwise, and the participant-specific missingness indicators are collected in $r_{x.j}=[r_{x.1j},\ldots ,r_{x.Tj}{]}^{\prime }$ .

The joint distribution of the observed and missing data, along with the missing data indicators, can be factorized into four components: the outcome model (the substantive model), the predictor model (the substantive model), the missingness model for the outcome, and the missingness model for the predictor (e.g., Jackson et al., 2010):

(1) $$\begin{array}{rl} & \mathrm{Pr}(y_{j},x_{j},r_{y.j},r_{x.j}|w_{j},\theta_{y},\theta_{x},\psi_{y},\psi_{x})\\ =\; & \mathrm{Pr}(y_{j}|x_{j},r_{x.j},w_{j},\theta_{y})\mathrm{Pr}(x_{j}|w_{j},\theta_{x})\mathrm{Pr}(r_{y.j}|y_{j},x_{j},r_{x.j},w_{j},\psi_{y})\mathrm{Pr}(r_{x.j}|x_{j},w_{j},\psi_{x}), \end{array}$$

where $w_{j}$ is a matrix of predictors, $\theta_{y}$ is the parameters of the outcome model, $\theta_{x}$ is the parameters of the predictor model, $\psi_{y}$ is the parameters of the missingness model for the outcome, and $\psi_{x}$ is the parameters of the missingness model for the predictor. The parameters $\theta_{y}$ and $\theta_{x}$ govern the outcome and predictor models, respectively, while $\psi_{y}$ and $\psi_{x}$ govern the missingness processes for the outcome and predictor variables. Consistent with the selection model assumption (Rubin, 1987), the parameters $[\theta_{y},\theta_{x},\psi_{y},\psi_{x}{]}^{\prime }$ are mutually independent. In Equation (1), the error terms in the substantive and missingness models are assumed to be uncorrelated by conditioning on appropriate predictors that account for the shared influences between the outcome and missingness process for the outcome, and between the predictor and missing process for the predictor. Within the outcome model—referred to as the “focal model”—the regression coefficients are the primary parameters of interest, while the predictor and two missing data models are considered “nuisance models” whose primary function is to ensure valid inference for the focal parameters.

**Figure 1:**
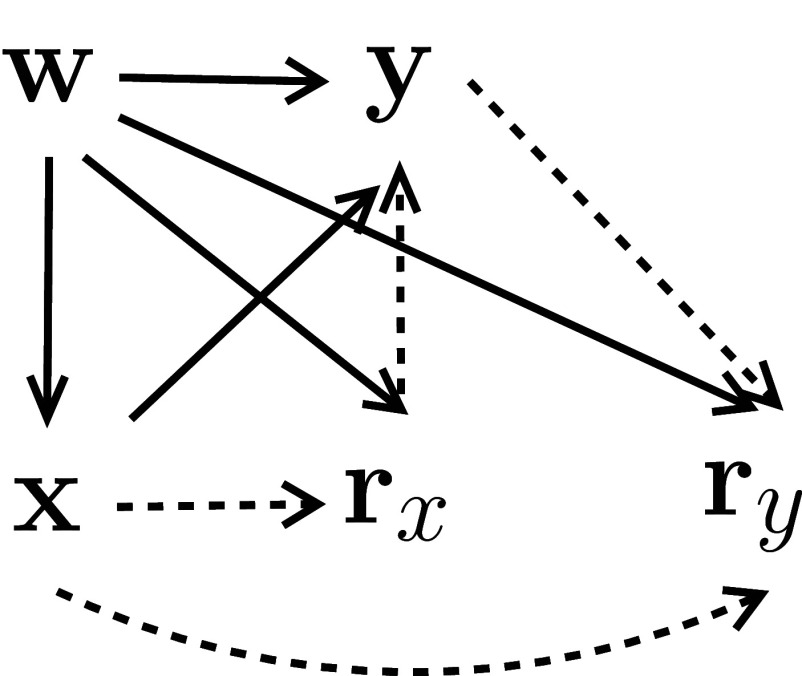
Directed acyclic graph of the joint model. *Note:* y is an outcome (suicidal behavior), x is a focal predictor (within-person daily interpersonal stress variable), w is a set of controlling predictors, $r_{y}$ is an indicator of missingness for the outcome (suicidal behavior), $r_{x}$ is an indicator of missingness for the predictor, the arrows represent conditional dependencies between these variables, and four dotted arrows are related to MNAR.

Figure 1 presents a directed acyclic graph (DAG; Pearl, 2009) illustrating the relationships among the variables in the joint model described by Equation (1). The DAG captures the dependencies among the outcome y , focal predictors x , and the indicators of missingness $r_{y}$ and $r_{x}$ . The arrows represent conditional dependencies between these variables, with w serving as a set of controlling predictors (except x ) that influence y , x , $r_{y}$ , and $r_{x}$ . The DAG also illustrates the diffuse MNAR process, wherein the probability of missingness in both $r_{y}$ and $r_{x}$ depends on both observed and missing data values (Gomer & Yuan, 2021). This aligns with the concept of indirect MNAR, which contrasts with focused MNAR (or direct MNAR), in which missingness depends only on the missing values themselves (Enders, 2022). The DAG visually encapsulates the joint modeling approach that accounts for these complex dependencies, supporting valid statistical inferences despite the presence of MNAR data. In Figure 1, four dotted arrows are related to MNAR. The arrow from y to $r_{y}$ indicates that the probability of y being missing depends directly on the value of y itself. Similarly, the arrow from x to $r_{x}$ shows that the missingness of x is directly determined by x ’s own value. The arrow from x to $r_{y}$ indicates that the missingness of y is influenced by the value of x , which means that the value of x contributes to the probability of y being missing. The arrow from $r_{x}$ to y shows that the outcome y is directly affected by whether x is missing, implying that the fact that x is missing is informative about y beyond what is captured by the observed values alone. Below, ϕ denotes the sensitivity parameters, defined as quantities that capture the dependence of the missingness mechanism on the unobserved values of partially observed outcomes or predictors, thereby representing departures from the MAR assumption. In Figure 1, the MAR assumption holds if the vector $[y,x{]}^{\prime }$ is conditionally independent of the missing indicators $[r_{y},r_{x}{]}^{\prime }$ , which is achieved by dropping the four dotted arrows.

For the selection of $w_{j}$ , including auxiliary variables—those associated with missingness but not the outcome—enhances model robustness and helps distinguish MNAR from MAR mechanisms, thereby improving the validity of sensitivity analyses (Little & Rubin, 2020). In addition, when an additional model, such as a predictor model ( $\mathrm{Pr}(x_{j}|w_{j},\theta_{x})$ ), is considered as an extension of a traditional selection model for outcomes only, the parameters in the predictor model are identifiable if a continuous predictor in the missingness model for outcomes ( $\mathrm{Pr}(r_{y.j}|y_{j},x_{j},r_{x.j},w_{j},\psi_{y})$ ), which does not appear in the predictor model, is included (Chen et al., 2018).

Below, each of the four components in Equation (1) is described, assuming that there is one time-varying predictor ( $x_{tj}$ ), as in our motivating example. For the research question discussed in Section 2, the within-level prior daily interpersonal stress variable (on day t−1 , denoted by $x_{(t-1)j}-x_{.j}$ , where $x_{.j}$ is the cluster (person) mean of $x_{tj}$ ) and the within-level daily interpersonal stress variable (on day *t*, denoted by $x_{tj}-x_{.j}$ ) are included in the model specification.

#### 3.1.1 Outcome model

A mixed-effects cloglog regression (e.g., Jiang, 2007) was chosen to model rare binary outcomes. In this specification, the latent error term is assumed to follow a right-skewed Gumbel (Type I extreme value) distribution for maxima, which induces the cloglog link. This choice is motivated by the distribution’s origins in extremes modeling and by the view that suicidal behaviors represent the high end of an underlying risk continuum. Relative to symmetric error models (logit/probit), the Gumbel’s asymmetric, heavier upper tail assigns more probability to upper-tail events while still concentrating mass on moderate values for most individuals, making it well-suited to rare, high-consequence outcomes plausibly precipitated by spikes in stress.

Specifically, a latent continuous response $y_{tj}^{*}$ is defined as follows:

(2) $$\begin{array}{rl} y_{tj}^{*} & =\eta_{tj}+e_{tj}\\ & =\gamma_{y.0}+\gamma_{y.1}(x_{(t-1)j}-x_{.j})+\gamma_{y.2}(x_{tj}-x_{.j})+\phi_{ry.j}(r_{x.tj}-r_{x..j})+\gamma_{y}^{⊤}w_{j}+u_{y.j}^{⊤}z_{y.tj}+e_{tj}, \end{array}$$

where $y_{tj}^{*}$ is the latent variable representing the underlying continuous process for the observed response for time *t* for person *j*, $r_{x..j}$ is the cluster (person) mean of $r_{x.tj}$ , $\eta_{tj}$ is the linear predictor, $e_{tj}$ is the Gumbel-distributed error, $\gamma_{y.0}$ is the fixed intercept, $\gamma_{y.1}$ is the fixed-effect coefficient for $x_{(t-1)j}-x_{.j}$ (focal parameter), $\gamma_{y.2}$ is the fixed-effect coefficient for $x_{tj}-x_{.j}$ (focal parameter), $\phi_{ry.j}$ is a subject-specific sensitivity parameter that captures the dependence between the missingness indicator $\left(r_{x.tj}-r_{x..j}\right)$ and the latent outcome $y_{tj}^{*}$ , $\gamma_{y}$ (excluding $\gamma_{y.0}$ , $\gamma_{y.1}$ , and $\gamma_{y.2}$ ) is a vector of fixed-effect coefficients associated with the predictors $w_{j}$ , $u_{y.j}$ is a vector of random effects for person *j*, and $z_{y.tj}$ is a matrix of random-effect predictors for time *t* for person *j*. The random effects are assumed to follow a multivariate normal distribution, $\mathrm{MVN}(0,\Sigma_{1})$ .

The Gumbel-based latent variable construction naturally leads to the cloglog link function for modeling the probability $\mathrm{Pr}(y_{tj}|x_{(t-1)j}-x_{.j},x_{tj}-x_{.j},r_{x.tj}-r_{x..j},w_{j},u_{y.j})$ as follows:

(3) $$\begin{array}{r} \mathrm{cloglog}[\mathrm{Pr}(y_{tj}=1|x_{(t-1)j}-x_{.j},x_{tj}-x_{.j},r_{x.tj}-r_{x..j},w_{j},u_{y.j})]=\eta_{tj.} \end{array}$$

Equivalently, the outcome probability under the cloglog model is expressed as

(4) $$\begin{array}{r} \mathrm{Pr}(y_{tj}=1|x_{(t-1)j}-x_{.j},x_{tj}-x_{.j},r_{x.tj}-r_{x..j},w_{j},u_{y.j})=1-\exp[-\exp(\eta_{tj})]. \end{array}$$

Compared to the logit or probit link functions, the cloglog link assumes that $\mathrm{Pr}(y_{tj}=1|x_{(t-1)j}-x_{.j},x_{tj}-x_{.j},r_{x.tj}-r_{x..j},w_{j},u_{y.j})$ approaches 0 fairly slowly but approaches 1 quite sharply.

The coefficients $\gamma_{y.0},\gamma_{y.1},\gamma_{y.2}$ , and the vector of coefficients $\gamma_{y}$ represent changes in the linear predictor $\eta_{tj}$ associated with one-unit increases in their corresponding predictors, holding all other variables constant. Under the cloglog link, the model admits a proportional hazards interpretation in discrete time. In the context of the daily diary data, this corresponds to the conditional probability that a participant experiences the outcome on a given day, given the predictors and random effects. Exponentiating a coefficient yields the *hazard ratio*, which represents the multiplicative change in this daily probability associated with a one-unit increase in the predictor. For example, a hazard ratio greater than 1 indicates that higher values of the predictor are associated with an increased conditional probability of experiencing the outcome on a given day, whereas a hazard ratio less than 1 indicates a decreased conditional probability.

#### 3.1.2 Time-varying predictor model

To model the probability of observing the presence of any interpersonal stressor on a given day ( $x_{tj}\in 0,1$ ), a probit mixed-effects model was used to link the latent propensity for stress occurrence to a linear predictor via the standard normal cumulative distribution function. A binary model for the time-varying predictor $x_{tj}$ can be specified using a probit link function as follows:

(5) $$\begin{array}{rl} \mathrm{Pr}(x_{tj}=1|w_{j},u_{x.j}) & =\mathrm{Pr}(x_{tj}^{*}>0|w_{j},u_{x.j})\\ & =\Phi (\gamma_{x.0}+\gamma_{x}^{⊤}w_{j}+u_{x.j}^{⊤}z_{x.tj}), \end{array}$$

where $x_{tj}^{*}$ is the latent variable representing the unobserved normal process underlying the observed binary variable $x_{tj}$ , $\gamma_{x.0}$ is a fixed intercept, $\gamma_{x}$ is a vector of fixed-effect coefficients associated with the person-level predictors $w_{j}$ , $u_{x.j}$ is a vector of person-specific random effects, $z_{x.tj}$ is a matrix of random-effect predictors, and Φ denotes the cumulative distribution function of the standard normal distribution, corresponding to the probit link function. The random effects are assumed to follow a multivariate normal distribution, $\mathrm{MVN}(0,\Sigma_{2})$ .

#### 3.1.3 Missing model for the outcome

The selection model (Heckman, 1976) allows the probability of non-response to depend directly on the outcome. In this model, the missing indicator is assumed to be a manifestation of a latent variable that may be associated with predictors. Diggle and Kenward (1994) expanded the Heckman model to continuous longitudinal data with informative drop-out (monotone missingness) within a general linear model framework. This approach was later widely extended to binary longitudinal data using a logistic regression model (e.g., Baker, 1995; Fitzmaurice et al., 1996). McNeish (2025) employed a within-level latent predictor for missingness (instead of using outcomes as in Diggle & Kenward [1994]) and introduced a random intercept to account for individual differences in missingness outcomes within a dynamic structural equation modeling framework. In this study, the missingness model is specified using within-level $y_{tj}-y_{.j}$ , similar to the Heckman probit selection model (Heckman, 1976), as a generalized mixed-effects model.

The conditional distribution of the missing response given the random effects $a_{y.j}$ is assumed to follow a Bernoulli distribution:

(6) $$\begin{array}{r} r_{y.tj}|a_{y.j}∼\text{Bernoulli}(1-\mathrm{Pr}(r_{y.tj}=0|y_{tj}-y_{.j},x_{tj}-x_{.j},w_{j},a_{y.j})), \end{array}$$

with a threshold latent variable model specified as follows:

(7) $$\begin{array}{r} r_{y.tj}^{*}=\omega_{y.0}+\phi_{y.j}(y_{tj}-y_{.j})+\phi_{xy.j}(x_{tj}-x_{.j})+\omega_{y}^{⊤}w_{j}+a_{y.j}^{⊤}z_{y.tj}+\nu_{y.tj}, \end{array}$$

where $r_{y.tj}^{*}$ is the latent variable representing the unobserved normal process underlying the binary missing outcome variable $r_{y.tj}$ , $\omega_{y.0}$ is the fixed intercept, $\omega_{y}$ (excluding $\omega_{y.0}$ ) is a vector of fixed-effect coefficients associated with the predictors $w_{j}$ , $\phi_{y.j}$ is a subject-specific sensitivity parameter that characterizes departures from the MAR assumption by capturing the dependence of the missingness mechanism on the outcome $\left(y_{tj}-y_{.j}\right)$ , $\phi_{xy.j}$ is a subject-specific sensitivity parameter that characterizes departures from the MAR assumption by capturing the dependence of the missingness mechanism on the predictor $\left(x_{tj}-x_{.j}\right)$ , $a_{y.j}$ is a vector of random effects for person *j*, $z_{y.tj}$ is a matrix of random-effect predictors for time *t* for person *j*, and $\nu_{y.tj}$ is the error term, assumed to follow a standard normal distribution for identifiability: $\nu_{tj}∼N(0,1)$ . The random effects are assumed to follow a multivariate normal distribution, $\mathrm{MVN}(0,\Sigma_{3})$ .

The latent Gaussian process leads to the following specification:

(8) $$\begin{array}{rl} \mathrm{Pr}(r_{y.tj}=0|y_{tj}-y_{.j},x_{tj}-x_{.j},w_{j},a_{y.j}) & =\mathrm{Pr}(r_{y.tj}^{*}>0|y_{tj}-y_{.j},x_{tj}-x_{.j},w_{j},a_{y.j})\\ & =\Phi (\omega_{y.0}+\phi_{y.j}(y_{tj}-y_{.j})+\phi_{xy.j}(x_{tj}-x_{.j})+\omega_{y}^{⊤}w_{j}+a_{y.j}^{⊤}z_{y.tj}). \end{array}$$

#### 3.1.4 Missing model for a time-varying predictor

A missingness model for a time-varying predictor can be specified similarly to the missingness model for the outcome:

(9) $$\begin{array}{rl} \mathrm{Pr}(r_{x.tj}=0|(x_{tj}-x_{.j}),w_{j},a_{x.j}) & =\mathrm{Pr}(r_{x.tj}^{*}>0|x_{tj}-x_{.j},w_{j},a_{x.j})\\ & =\Phi (\omega_{x.0}+\phi_{x.j}(x_{tj}-x_{.j})+\omega_{x}^{⊤}w_{j}+a_{x.j}^{⊤}z_{x.tj}), \end{array}$$

where $r_{x.tj}^{*}$ is the latent variable representing the unobserved normal process underlying the binary missing outcome variable $r_{x.tj}$ , $\omega_{x.0}$ is the fixed intercept, $\omega_{x}$ (excluding $\omega_{x.0}$ ) is a vector of fixed-effect coefficients associated with the predictors $w_{j}$ , $\phi_{x.j}$ is a subject-specific sensitivity parameter that characterizes departures from the MAR assumption by capturing the dependence of the missingness mechanism on the predictor $\left(x_{tj}-x_{.j}\right)$ , $a_{x.j}$ is a vector of random effects for person *j*, and $z_{x.tj}$ is a matrix of random-effect predictors for time *t* for person *j*. The random effects are assumed to follow a multivariate normal distribution, $\mathrm{MVN}(0,\Sigma_{4})$ .

### 3.2 Parameter estimation

The specified selection model involves a large number of parameters, and missing observations would need to be integrated out of the likelihood, making the direct maximization of the resulting likelihood a non-trivial task. Thus, Markov chain Monte Carlo (MCMC) sampling was conducted using WinBUGS.^1^ WinBUGS is a Windows-based software package for Bayesian inference using MCMC methods. It relies primarily on Gibbs sampling, with automatic implementation of conjugate updating where applicable. WinBUGS supports a model specification language that allows flexible formulation of hierarchical Bayesian models and incorporates adaptive techniques such as block updating and slice sampling to improve mixing and convergence. The software also facilitates data augmentation for handling missing data and discrete latent variables, making it well-suited for the model specified in the current study.

Data augmentation (e.g., Tanner & Wong, 1987) is used to construct the likelihood. Specifically, the observed outcome $y_{tj}^{o}$ is augmented with the missing outcomes $y_{tj}^{m}$ , forming $y_{tj}=[y_{tj}^{o},y_{tj}^{m}{]}^{\prime }.$ Similarly, the observed predictor $x_{tj}^{o}$ is augmented with the missing predictor $x_{tj}^{m}$ , forming $x_{tj}=[x_{tj}^{o},x_{tj}^{m}{]}^{\prime }.$ With these augmentations, the complete-data likelihood of the parameters $\Theta =[\gamma_{y.0},\gamma_{y.1},\gamma_{y.2},\gamma_{y},\phi_{ry.j},u_{y.j},\gamma_{x.0},\gamma_{x},u_{x.j},\omega_{y.0},\phi_{y.j},\phi_{xy.j},\omega_{y},a_{y.j},\omega_{x.0},\omega_{x},\phi_{x.j},a_{x.j}{]}^{\prime }$ can be written as follows:

(10) $$\begin{array}{rl} L(\Theta ) & =\prod_{j=1}^{J}[\prod_{t=1}^{T}\{\\ & \underset{\text{Outcome Model}}{\underbrace{\mathrm{Pr}(y_{tj}=1|x_{(t-1)j}-x_{.j},x_{tj}-x_{.j},r_{x.tj}-r_{x..j},w_{j},u_{y.j}{)}^{\;y_{tj}}\;\mathrm{Pr}(y_{tj}=0|x_{(t-1)j}-x_{.j},x_{tj}-x_{.j},r_{x.tj}-r_{x..j},w_{j},u_{y.j}{)}^{\;1-y_{tj}}}}\\ & \;\times \;\underset{\text{Predictor Model}}{\underbrace{\mathrm{Pr}(x_{tj}=1|w_{j},u_{x.j}{)}^{\;x_{tj}}\;\mathrm{Pr}(x_{tj}=0|w_{j},u_{x.j}{)}^{\;1-x_{tj}}}}\\ & \;\times \;\underset{\text{Missing Model for Outcome}}{\underbrace{\mathrm{Pr}(r_{y.tj}=1|y_{tj}-y_{.j},x_{tj}-x_{.j},w_{j},a_{y.j}{)}^{\;r_{y.tj}}\;\mathrm{Pr}(r_{y.tj}=0|y_{tj}-y_{.j},x_{tj}-x_{.j},w_{j},a_{y.j}{)}^{\;1-r_{y.tj}}}}\\ & \;\times \;\underset{\text{Missing Model for Predictor}}{\underbrace{\mathrm{Pr}(r_{x.tj}=1|x_{tj}-x_{.j},w_{j},a_{x.j}{)}^{\;r_{x.tj}}\;\mathrm{Pr}(r_{x.tj}=0|x_{tj}-x_{.j},w_{j},a_{x.j}{)}^{\;1-r_{x.tj}}}}\}]. \end{array}$$

Posterior inference is conducted using an MCMC algorithm within this data augmentation framework. At each iteration, missing values are treated as latent variables and are sampled from their full conditional distributions given the observed data and current parameter values. Conditional on the augmented (completed) data, model parameters are then updated from their corresponding full conditional distributions. This iterative procedure corresponds to Bayesian data augmentation, in which imputation and parameter estimation are performed jointly. Because missing values are repeatedly sampled across MCMC iterations, uncertainty due to missing data is naturally propagated into posterior inference, avoiding the underestimation of variability that may arise under single imputation approaches (e.g., Du et al., 2022).

Prior distributions for the parameters are specified as follows. As assumed in the joint model, *a priori* independence was imposed between the parameters of the outcome model and the missingness model for the outcome, as well as between the parameters of the predictor model and the missingness model for the predictor. For the fixed parameters ( $\gamma_{y.0},\gamma_{y.1},\gamma_{y.2},\gamma_{y},\gamma_{x.0},\gamma_{x},\omega_{y.0},\omega_{y},\omega_{x.0},\omega_{x}$ ), a normal distribution was assumed, with a mean of 0 and a variance of 5. For the random parameters ( $u_{y.j},a_{y.j},u_{x.j},a_{x.j}$ ), a multivariate normal distribution was assumed when both a random intercept and random slopes were included in the model, with an inverse-Wishart prior specified for the variance–covariance matrix ( $W^{-1}(I_{K},K+1)$ ), where *K* is the number of random effects. When only a random intercept was included, it was assumed to follow a normal distribution with mean 0, and an inverse-gamma prior was specified for its variance, with a mean of 1 and variance of 10. When a first-order lag time-varying predictor, $\left(x_{tj}-x_{.j}\right)$ , was included, $\left(x_{(t-1)j}-x_{.j}\right)$ at t=1 was assumed to follow a normal distribution, where the mean follows a normal prior with mean 0 and variance 5, and the variance follows an inverse-gamma prior with mean 1 and variance 10.

(11) $$\begin{array}{r} \phi_{.j}∼N(\mu_{\phi },\sigma_{\phi });\mu_{\phi }∼N(0,5),\sigma_{\phi }∼\text{HalfCauchy}(0,1.5). \end{array}$$

Using the weakly informative priors allowed us to validate the robustness of the findings while acknowledging the uncertainty in the precise values of the sensitivity parameters.

Because the sensitivity parameters $[\phi_{y.j},\phi_{x.j},\phi_{xy.j},\phi_{ry.j}{]}^{\prime }$ relating to MNAR cannot be directly estimated from the observed data alone, they are not identifiable without additional, typically untestable assumptions about the missing data mechanism (Daniels & Hogan, 2008; Little & Rubin, 2020; Molenberghs & Kenward, 2007). Such assumptions may involve fixing these parameters at prespecified values or specifying informative prior distributions in a Bayesian framework. In the Bayesian context, sensitivity analysis can be conducted by examining a range of prior specifications for the sensitivity parameters (e.g., Hogan et al., 2014), including informative, weakly informative, and diffuse priors. In preliminary analyses, alternative prior specifications were explored. However, highly diffuse priors led to unstable estimation and convergence difficulties, likely due to the non-identifiability of the sensitivity parameters. In addition, specifying informative priors was not feasible in this application because no substantive prior knowledge was available to justify particular parameter values. Therefore, weakly informative priors were adopted for the sensitivity parameters, providing regularization while avoiding undue influence on posterior estimates. The weakly informative priors were specified for the sensitivity parameters:

(12) $$\begin{array}{r} \phi_{.j}∼N(\mu_{\phi },\sigma_{\phi });\;\mu_{\phi }∼N(0,5),\;\sigma_{\phi }∼\text{HalfCauchy}(0,1.5). \end{array}$$

Using these weakly informative priors allows the robustness of the findings to be assessed while acknowledging uncertainty in the sensitivity parameters.

Convergence was evaluated with three chains using the Gelman–Rubin statistic (Gelman & Rubin, 1992). When the Gelman–Rubin statistic is sufficiently close to 1, it indicates convergence. The recommended cutoff for acceptable convergence is 1.1 (Gelman et al., 2013).

### 3.3 Posterior predictive model checking

Posterior predictive checking (PPC; Gelman et al., 1996) was conducted to assess the adequacy of the model in capturing key features of the observed data. This was achieved by comparing observed test statistics to their posterior predictive distributions. The posterior predictive *p*-value ( ppp -value) quantifies the proportion of replicated test statistics from the posterior predictive distribution that are greater than or equal to the observed test statistic.

For the substantive models, the test statistics were (a) the proportion of positive outcomes among participants who exhibited suicidal behavior and (b) the proportion of days with endorsement of daily interpersonal stress (i.e., $x_{tj}=1$ ) for each participant *j*:

(13) $$\begin{array}{r} T_{y}(y_{j})=\frac{\sum_{t=1}^{n_{j}}y_{tj}}{T},\;T_{x}(x_{j})=\frac{\sum_{t=1}^{n_{j}}x_{tj}}{T}. \end{array}$$

For the missingness models, the test statistics were the overall observed rates for the outcome and predictor:

(14) $$\begin{array}{r} T_{r_{y}}(r_{y.j})=\frac{\sum_{t=1}^{n_{j}}r_{y.tj}}{T},\;T_{r_{x}}(r_{x.j})=\frac{\sum_{t=1}^{n_{j}}r_{x.tj}}{T}. \end{array}$$

Posterior samples of the parameters were drawn from the fitted model. For each posterior sample, replicated data $y^{\text{rep}},\;x^{\text{rep}},\;r_{y}^{\text{rep}},\text{and}\;r_{x}^{\text{rep}}$ were generated. The replicated test statistics $T_{y}^{\text{rep}},\;T_{x}^{\text{rep}},\;T_{r_{y}}^{\text{rep}},\;\text{and}\;T_{r_{x}}^{\text{rep}}$ were computed for each posterior draw. Posterior predictive *p*-values were calculated as

(15) $$\begin{array}{r} ppp_{y}=\frac{1}{S}\sum_{s=1}^{S}I\left(T_{y}^{\text{rep}(s)}\geq T_{y}(y_{j})\right),\;ppp_{x}=\frac{1}{S}\sum_{s=1}^{S}I\left(T_{x}^{\text{rep}(s)}\geq T_{x}(x_{j})\right), \end{array}$$

(16) $$\begin{array}{r} ppp_{r_{y}}=\frac{1}{S}\sum_{s=1}^{S}I\left(T_{r_{y}}^{\text{rep}(s)}\geq T_{r_{y}}(r_{y.j})\right),\;ppp_{r_{x}}=\frac{1}{S}\sum_{s=1}^{S}I\left(T_{r_{x}}^{\text{rep}(s)}\geq T_{r_{x}}(r_{x.j})\right), \end{array}$$

where I[⋅] is the indicator function, and *S* is the number of posterior samples after burn-in. Values of ppp close to 0.5 indicate good model fit, whereas values near 0 or 1 suggest lack of fit.

Quantiles (5th, 50th, and 95th) of the posterior predictive distributions were computed for the replicated test statistics ( $T_{y}^{\text{rep}},T_{x}^{\text{rep}},T_{r_{y}}^{\text{rep}},$ and $T_{r_{x}}^{\text{rep}}$ ) to summarize the range of plausible values under the fitted model. Visualizations were used to compare the observed test statistics ( $T_{y}(y_{j}),T_{x}(x_{j}),T_{r_{y}}(r_{y.j}),$ and $T_{r_{x}}(r_{x.j})$ ) to their posterior predictive distributions. Observed frequencies were represented as points, while the posterior predictive medians (50th percentiles) were plotted as lines. If the 95% posterior predictive intervals for the replicated statistics included the observed statistics, the model was considered to adequately reproduce the observed data.

## 4 Illustration

In this section, the models specified in Section 3 are applied to the empirical dataset presented in Section 2.

### 4.1 Predictor selection and processing

Predictor selection for the outcome, predictor, and missing data models was guided by an extensive literature review conducted by domain experts in clinical psychology. The final set of baseline, person-level, and time-varying predictors was identified to reflect known risk and protective factors for suicidal behavior, as well as variables associated with reporting patterns and stressor exposure. As summarized in Appendix C, multiple studies across clinical and developmental psychopathology support the inclusion of variables, such as baseline suicidal ideation severity, past suicide attempts, demographic factors (age, sex, and sexual minority status), trauma history, peer victimization, internalizing symptoms (depression and anxiety), coping flexibility, parental and peer/friend support, and sleep. Time-varying predictors—including daily interpersonal stress (focal predictor), survey day (to capture trend effects), and calendar-based features (i.e., weekends and summer months)—were selected based on their theoretical relevance and empirical associations with day-to-day variation in suicidal behavior and data observability. Moreover, predictors were included in the corresponding missingness models for the outcome (suicidal behavior) and the predictor (daily interpersonal stress) when prior findings supported nonrandom patterns of nonresponse. This structured approach ensures that model specification was informed by substantive theory and empirical evidence, enhancing interpretability and construct validity. None of the predictors exhibited high collinearity (i.e., all absolute Pearson correlation coefficients were below 0.80).

As summarized in Appendix A, missingness for sexual-minority and gender-minority indicators is 5%, and missingness among baseline predictors ranges from 5.7% (suicidal ideation severity) to 17% (peer-victimization scales). To address missing data in these predictors, multiple imputation was conducted using the mice package in R (van Buuren & Groothuis-Oudshoorn, 2011) with random forest as the imputation method. The random forest method was selected for its nonparametric nature and flexibility in handling nonlinear associations and mixed data types. The differences in means and standard deviations before and after imputation were minimal, suggesting that the procedure preserved the original distributional properties of the data.

### 4.2 Analysis

Cluster-mean centering at the participant level of the focal time-varying variable (daily interpersonal stress) was used to disaggregate within-participant (day-level) effects from between-person effects (e.g., Raudenbush & Bryk, 2002). Because daily interpersonal stress contained missing values, a selection model was employed to jointly model the observed responses and the missingness mechanism within a Bayesian framework, enabling imputation during MCMC sampling. Accordingly, the person-level cluster mean of daily interpersonal stress—used to define the between-person component—was updated at each MCMC iteration based on both observed and imputed values. This procedure ensures that the within- and between-participant decompositions appropriately reflect uncertainty due to missingness and remain fully integrated with the joint model. In contrast, the three other time-varying predictors—survey day, weekends, and summer months—contained no missing values and were also cluster-mean centered.

To determine the appropriate functional form between participant-level continuous predictors and the cluster means of outcomes (suicidal behavior, suicidal behavior missingness, daily interpersonal stress, and daily interpersonal stress missingness), the participant-level outcome (i.e., the empirical logit of the proportion) was plotted against each participant-level predictor. Each plot included locally weighted scatterplot smoothing (LOWESS), linear, quadratic, and cubic trend lines to visually assess model fit. Linear terms were retained when the linear and LOESS trends were closely aligned, indicating negligible nonlinearity. In cases where LOESS trends showed marked curvature or inflection, quadratic or cubic terms were used to better capture the underlying relationship. This visual diagnostic approach supported the selection of functional forms that adequately reflected observed trends without overfitting. Most predictors demonstrated linear associations across outcomes, indicating adequacy of linear specification. However, several predictors exhibited outcome-specific nonlinearity, warranting the inclusion of quadratic terms (see the added quadratic terms in Tables 1 and 2). Categorical predictors were dummy-coded.

**Table 1:** Empirical study: Results of the outcome Table 1 long description.

		MNAR-cloglog			MAR-cloglog			MNAR-logit	
**Outcome**		Median	CrI		Median	CrI		Median	CrI
*Substantive model*									
Intercept[ $\gamma_{y.0}$ ]		−0.898	[−2.635,0.789]		−7.036	[−8.726,−5.659]		−0.109	[−2.110,2.150]
Prior stress within[ $\gamma_{y.1}$ ]		0.374	[−0.225,1.090]		0.943	[−0.550,2.006]		0.245	[−0.484,1.017]
Current stress within[ $\gamma_{y.2}$ ]		1.114	[0.309,1.940]		1.754	[0.760,2.812]		1.199	[0.419,2.028]
Suicidal ideation severity[ $\gamma_{y.3}$ ]		−0.249	[−1.448,0.789]		0.569	[−0.199,1.508]		−0.118	[−1.424,1.110]
Num. of past suicide attempts[ $\gamma_{y.4}$ ]		0.760	[−0.423,2.811]		−0.532	[−1.610,0.803]		−0.125	[−2.348,2.170]
Quadratic of num. of past suicide attempts[ $\gamma_{y.5}$ ]		−0.176	[−0.674,0.171]		0.277	[−0.033,0.491]		0.038	[−0.496,0.631]
Interview age[ $\gamma_{y.6}$ ]		−0.180	[−1.211,0.819]		0.468	[−0.308,1.545]		−0.114	[−1.327,0.998]
Quadratic of interview age[ $\gamma_{y.7}$ ]		−0.107	[−0.916,0.712]		−0.027	[−0.576,0.421]		−0.183	[−1.104,0.647]
Sex[ $\gamma_{y.8}$ ]		−0.973	[−2.343,0.749]		−0.919	[−2.532,0.640]		−0.749	[−2.769,1.329]
Gender minority[ $\gamma_{y.9}$ ]		0.210	[−2.615,2.577]		0.032	[−1.625,1.839]		−0.076	[−2.714,2.506]
Sexual minority[ $\gamma_{y.10}$ ]		−1.308	[−3.306,0.688]		−0.396	[−1.854,1.301]		−1.507	[−4.116,0.748]
Trauma history[ $\gamma_{y.11}$ ]		0.840	[−0.097,1.847]		−0.116	[−0.918,0.658]		1.068	[−0.168,2.177]
Peer victimization[ $\gamma_{y.12}$ ]		1.724	[0.419,3.015]		0.770	[0.008,1.780]		1.973	[0.583,3.404]
Relational peer victimization[ $\gamma_{y.13}$ ]		−0.843	[−2.088,0.413]		−0.285	[−1.265,0.689]		−0.784	[−2.144,0.843]
Reputational relational peer victimization[ $\gamma_{y.14}$ ]		0.150	[−1.059,1.090]		−0.680	[−1.812,0.311]		−0.050	[−1.653,1.739]
Parental support[ $\gamma_{y.15}$ ]		−0.405	[−1.359,0.525]		−0.505	[−1.212,0.272]		−0.360	[−1.411,0.894]
Survey day[ $\gamma_{y.16}$ ]		0.007	$\left[1.107\times 10^{-4},0.016\right]$		0.004	[−0.013,0.024]		0.007	[−0.006,0.019]
SD of intercept[SD of $u_{y.0}$ ]		4.069	[3.160,5.180]		1.321	[0.316,2.622]		5.200	[4.179,6.434]
Mean of MNAR effect for $r_{x}$ [ $\mu_{ry.\phi }$ ]		−15.080	[−17.430,−12.620]		-			−18.110	[−20.530,−15.890]
SD of MNAR effect for $r_{x}$ [ $\sigma_{ry.\phi }$ ]		3.642	[1.367,6.595]		-			4.062	[1.760,6.461]
*Missing model*									
Intercept[ $\omega_{y.0}$ ]		−1.701	[−2.962,−0.538]		1.706	[1.474,1.909]		−1.829	[−3.253,−0.613]
Suicidal ideation severity[ $\omega_{y.1}$ ]		−0.265	[−0.663,0.075]		0.048	[−0.017,0.113]		−0.275	[−0.714,0.087]
Quadratic of suicidal ideation severity[ $\omega_{y.2}$ ]		0.142	[−0.145,0.455]		−0.049	[−0.105,0.007]		0.139	[−0.170,0.496]
Trauma history[ $\omega_{y.3}$ ]		0.139	[−0.133,0.441]		0.059	[0.004,0.117]		0.156	[−0.134,0.481]
Parental support[ $\omega_{y.4}$ ]		0.329	[0.039,0.646]		0.001	[−0.058,0.059]		0.351	[0.043,0.700]
NSSI[ $\omega_{y.5}$ ]		0.140	[−0.842,1.018]		0.114	[−0.038,0.299]		0.135	[−1.000,1.069]
Summer within[ $\omega_{y.6}$ ]		−0.487	[−2.820,1.460]		−2.680	[−3.279,−2.203]		−0.439	[−2.791,1.549]
Summer between[ $\omega_{y.7}$ ]		−0.038	[−2.650,2.341]		−2.205	[−2.915,−1.569]		0.044	[−2.691,2.532]
Weekend within[ $\omega_{y.8}$ ]		−3.372	[−4.559,−2.356]		−2.975	[−3.455,−2.598]		−3.470	[−4.782,−2.388]
Weekend between[ $\omega_{y.9}$ ]		−4.574	[−7.303,−1.849]		−13.600	[−14.450,−12.810]		−4.398	[−7.296,−1.321]
SD of random intercept[SD of $a_{y.j}$ ]		0.435	[0.198,0.813]		0.220	[0.174,0.275]		0.468	[0.182,0.960]
Mean of MNAR effect for *y*[ $\mu_{y.\phi }$ ]		8.031	[7.181,9.502]		-			8.613	[6.955,9.851]
Mean of MNAR effect for *x*[ $\mu_{xy.\phi }$ ]		−0.663	[−1.155,0.165]		-			−0.719	[−1.227,0.142]
SD of MNAR effect for *y*[ $\sigma_{y.\phi }$ ]		0.359	[0.053,1.372]		-			0.565	[0.167,1.501]
SD of MNAR effect for *x*[ $\sigma_{xy.\phi }$ ]		0.497	[0.076,1.238]		-			0.496	[0.025,1.408]

*Note*: Bold indicates significance of fixed effects based on the 95% credible interval (CrI); - indicates that the effect was not modeled.

**Table 2:** Empirical study: Results of the predictor Table 2 long description.

		MNAR-cloglog			MAR-cloglog			MNAR-logit	
**Predictor**		Median	CrI		Median	CrI		Median	CrI
*Substantive model*									
Intercept[ $\gamma_{x.0}$ ]		−1.339	[−1.623,−0.955]		−1.222	[−1.648,−0.891]		−1.313	[−1.700,−1.047]
Suicidal ideation severity[ $\gamma_{x.1}$ ]		−0.022	[−0.260,0.213]		−0.023	[−0.270,0.197]		−0.048	[−0.250,0.175]
Interview age[ $\gamma_{x.2}$ ]		0.009	[−0.171,0.191]		0.016	[−0.183,0.217]		0.034	[−0.157,0.183]
Sex[ $\gamma_{x.3}$ ]		0.356	[−0.116,0.741]		0.259	[−0.112,0.797]		0.305	[0.018,0.731]
Gender minority[ $\gamma_{x.4}$ ]		−0.308	[−0.849,0.203]		−0.417	[−0.907,0.174]		−0.326	[−0.812,0.148]
Sexual minority[ $\gamma_{x.5}$ ]		0.273	[−0.218,0.629]		0.327	[−0.074,0.733]		0.318	[−0.113,0.683]
Parental support[ $\gamma_{x.6}$ ]		−0.134	[−0.327,0.072]		−0.132	[−0.326,0.077]		−0.123	[−0.328,0.045]
Depression symptoms[ $\gamma_{x.7}$ ]		0.278	[−0.128,0.689]		0.254	[−0.170,0.729]		0.319	[−0.041,0.746]
Anxiety symptoms[ $\gamma_{x.8}$ ]		0.254	[−0.051,0.551]		0.270	[−0.050,0.533]		0.211	[−0.051,0.547]
Coping flexibility - multiple coping[ $\gamma_{x.9}$ ]		0.013	[−0.232,0.245]		−0.048	[−0.285,0.244]		−0.009	[−0.233,0.336]
Coping flexibility - situational coping[ $\gamma_{x.10}$ ]		0.116	[−0.117,0.339]		0.151	[−0.064,0.374]		0.141	[−0.137,0.364]
Coping flexibility - coping rigidity[ $\gamma_{x.11}$ ]		−0.068	[−0.262,0.115]		−0.042	[−0.221,0.123]		−0.065	[−0.234,0.114]
Peer/friend support[ $\gamma_{x.12}$ ]		0.054	[−0.133,0.238]		0.056	[−0.143,0.244]		0.068	[−0.126,0.244]
Sleep disturbance[ $\gamma_{x.13}$ ]		−0.087	[−0.319,0.168]		−0.057	[−0.318,0.186]		−0.101	[−0.343,0.146]
Sleep impairment[ $\gamma_{x.14}$ ]		−0.077	[−0.333,0.172]		−0.118	[−0.352,0.110]		−0.079	[−0.351,0.172]
SD of random intercept[SD of $u_{x.0}$ ]		0.782	[0.645,0.962]		0.821	[0.680,1.013]		0.769	[0.637,0.948]
*Missing model*									
Intercept[ $\omega_{x.0}$ ]		1.851	[1.715,1.983]		1.818	[1.684,1.950]		1.858	[1.716,2.005]
Suicidal ideation severity[ $\omega_{x.1}$ ]		0.054	[−0.009,0.118]		0.043	[−0.017,0.105]		0.054	[−0.008,0.114]
Summer within[ $\omega_{x.2}$ ]		−3.127	[−3.982,−2.510]		−3.061	[−3.820,−2.456]		−3.137	[−3.951,−2.505]
Summer between[ $\omega_{x.3}$ ]		−2.662	[−3.577,−1.932]		−2.577	[−3.401,−1.846]		−2.683	[−3.586,−1.907]
Weekend within[ $\omega_{x.4}$ ]		−3.232	[−3.808,−2.778]		−3.186	[−3.726,−2.745]		−3.245	[−3.857,−2.782]
Weekend between[ $\omega_{x.5}$ ]		−14.400	[−15.350,−13.540]		−14.180	[−15.010,−13.290]		−14.450	[−15.490,−13.520]
SD of intercept[SD of $a_{x.j}$ ]		0.223	[0.168,0.286]		0.233	[0.186,0.290]		0.224	[0.173,0.286]
Mean of MNAR effect for *x*[ $\mu_{x.\phi }$ ]		−0.150	[−0.410,0.076]		-			−0.169	[−0.439,0.063]
SD of MNAR effect for *x*[ $\sigma_{x.\phi }$ ]		0.223	[0.168,0.286]		-			0.523	[0.142,0.876]

*Note*: Bold indicates significance of fixed effects based on the 95% credible interval (CrI); - indicates that the effect was not modeled.

When a random slope for the within-level daily interpersonal stress predictor was included in the Bayesian selection model, convergence issues arose in estimating the variance of the random slopes, which may indicate potential overfitting. Therefore, only a fixed effect for the variable was considered.

Following recommendations in the literature for the selection model (e.g., Daniels & Hogan, 2008; Molenberghs & Kenward, 2007), different assumptions regarding the missingness mechanism—specifically, MNAR and MAR, were examined to explore how varying these assumptions affects the conclusions of our primary research question as part of a sensitivity analysis. In the model under MAR, the sensitivity parameters $[\phi_{y.j},\;\phi_{x.j},\;\phi_{xy.j},\;\phi_{ry.j}{]}^{\prime }$ were set to 0, yielding a restricted version of the joint model in which the dependence between missingness and unobserved values is removed. This MAR-cloglog specification serves as the operational comparator to approaches that handle missingness without explicitly modeling MNAR mechanisms, including commonly used separate-model strategies. Because such approaches either rely on MAR-based imputation or ignore missingness entirely, they are statistically equivalent to this restricted model. In contrast, the full MNAR specification allows the dependence between missingness and unobserved outcomes or predictors to be parameterized through the sensitivity parameters. In addition, the selection model using the logit link, instead of the cloglog link function used in the outcome model, was fit for comparison purposes.

There were no convergence problems when fitting the two selection models—one assuming MNAR and the other assuming MAR—using the cloglog link function (named MNAR-cloglog and MAR-cloglog, respectively), as well as the MNAR model using the logit link (MNAR-logit).^2^ The Gelman–Rubin statistic ranged from 0.99 to 1.11 for all parameters. A conservative burn-in of 4,000 iterations was used, followed by 8,000 post-burn-in iterations for each model. Thinning was not applied, as autocorrelations in the sampled values were low. Monte Carlo standard error (MCSE) was examined to assess post-burn-in convergence, with MCSE values less than 0.01 times the posterior standard deviation.

### 4.3 Results

Tables 1 and 2 present the results of the MNAR-cloglog, MAR-cloglog, and MNAR-logit models for the outcome and predictor models, respectively. Below, the significance of each fixed effect in the MNAR-cloglog model is interpreted, followed by an evaluation of whether key parameter estimates, confidence intervals, and substantive conclusions change meaningfully when moving from the MAR to the MNAR scenario. Subsequently, the results from the MNAR-cloglog and MAR-logit models are compared.

#### 4.3.1 Results of MNAR-cloglog

For the MNAR-cloglog model, the resulting ppp values ranged from 0.329 to 0.723, indicating that the selection model adequately captures the observed proportions of positive outcomes and missingness patterns. Appendix D shows posterior predictive checks for the selection model, with the top panel corresponding to the outcome model and the bottom panel to the predictor model. In both panels, blue dots represent observed person-level proportions, red triangles indicate posterior median predictions, and red error bars denote 95% credible intervals (CrIs). The alignment between observed proportions and model predictions across most individuals suggests that the selection model adequately reproduces the empirical data under the specified missing data mechanism. Wider CrIs among participants with extreme or sparse endorsement rates reflect increased posterior uncertainty. Overall, the close agreement between observed and predicted values supports the model’s capacity to capture individual-level variation. However, it is important to note that evidence of good model fit does not confirm the correctness of the assumed missingness mechanism, as different mechanisms can produce similar observed data patterns (e.g., Molenberghs et al., 2008). Nevertheless, presenting these results is essential to demonstrate that the model is capable of reproducing key features of the observed data, which is a prerequisite for drawing valid inferences under the assumed missingness mechanism.

For the substantive model of the outcome (suicidal behavior) in Table 1, the effect regarding a primary research question—the within-person daily interpersonal stress—was significantly associated with suicidal behavior ( ${\hat{\gamma }}_{y.2}=1.114$ , 95% CrI = [0.309,1.940] ). This indicates a hazard ratio of exp(1.114)≈3.05 , which corresponds to a 205% ( (3.05−1)×100 ) increase in the hazard of suicidal behavior for each one-unit increase in daily interpersonal stress, holding the other predictors constant. The 95% CrI for the hazard ratio, approximately [exp(0.309),exp(1.940)]≈[1.36,6.96] , does not include one. However, the within-person prior daily interpersonal stress was not significantly associated with suicidal behavior ( exp(0.374)≈1.45,(1.45−1)×100≈45% , CrI = [−0.225,1.090]] ). In addition, the peer victimization measure, which assesses peer victimization through physical and verbal aggression, was significantly associated with suicidal behavior ( ${\hat{\gamma }}_{y.12}=1.724,95\%$ CrI = [0.419,3.015] ). The hazard ratio for a one-SD increase in direct victimization was estimated at 5.607 ( exp(1.724) ), with a 95% CrI of approximately [1.520,20.389] . This indicates that the hazard of suicidal behavior is approximately 461% ( exp(1.724)≈5.61,(5.61−1)×100 ) higher, holding other predictors constant. Furthermore, the effect of day (cluster-mean centered) was estimated as 0.007 ( 95% CrI = $\left[1.107\times 10^{-4},0.016\right]$ ), indicating that relative to an individual’s own mean observation day, each additional day was associated with a 0.70% increase in the hazard ( exp(0.007)−1≈0.007 ). Over a 90-day period, this corresponds to an estimated hazard ratio of 1.88 ( exp(0.007)×90 ), meaning the hazard of the event occurring at the end of the 90-day period is approximately 88% higher than at the beginning of the period, when comparing within the same individual.

For the missing model of the outcome (suicidal behavior) in Table 1, parental support was positively associated with data availability ( ${\hat{\omega }}_{y.4}=0.329$ , CrI = [0.039,0.646] ). Under the probit link, this coefficient is expressed on the latent *z*-score scale. Assuming a baseline latent score of 0 (corresponding to a probability of 0.50 ), the slope of the standard normal CDF at this point is ϕ(0)≈0.3989 . Multiplying this slope by the coefficient gives Δp≈0.3989×0.329≈0.131 , indicating that a one-unit increase in parental support is associated with approximately a 13-percentage-point increase in the probability of having observed suicidal behavior data, holding other predictors constant. In addition, the within-person effect of weekends indicates that, on weekends relative to weekdays, participants were significantly more likely to provide observed data, as reflected by a −3.372 unit decrease in the latent *z*-score for missingness (CrI = [−4.559,−2.356] ), holding all other predictors constant. For reference, a coefficient of −3.372 under the probit link corresponds to a decrease in the probability of missingness from 0.50 to Φ(−3.372)≈0.00037 , meaning the probability of observed data increases to about 0.99963 . Furthermore, the between-person effect showed that individuals with a higher overall proportion of weekend days in their study period were also more likely to provide observed data, with a −4.574 unit decrease in the latent *z*-score for missingness (CrI = [−7.303,−1.849] ) associated with greater weekend exposure, after accounting for within-person variability and other predictors. A coefficient of −4.574 corresponds to $\Phi (-4.574)\approx 2.4\times 10^{-6}$ for missingness, meaning the probability of observed data is effectively 1.00 .

For the substantive model of the predictor (daily interpersonal stress) in Table 2, no predictors were significant. For the missing model of the predictor in Table 2, participants were significantly more compliant on summer days ( ${\hat{\omega }}_{x.2}=-3.127$ , CrI = [−3.982,−2.510] ) and weekends ( ${\hat{\omega }}_{x.4}=-3.232$ , CrI = [−3.808,−2.778] ) compared to other days, with large decreases in the latent *z*-score for missingness. For reference, under the probit link, a coefficient of −3.127 corresponds to a change in the probability of missingness from 0.50 to Φ(−3.127)≈0.00088 , meaning the probability of having observed daily interpersonal stress data increases to about 0.99912 . Similarly, a coefficient of −3.232 corresponds to Φ(−3.232)≈0.00061 for missingness, implying a probability of observed data of about 0.99939 . Moreover, individuals sent daily survey links over more summer and weeekend days ( ${\hat{\omega }}_{x.3}=-2.662$ , CrI = [−3.577,−1.932] ) ( ${\hat{\omega }}_{x.5}=-14.400$ , CrI = [−15.350,−13.540] ) demonstrated overall better compliance, controlling for the other predictors. For example, Φ(−2.662)≈0.00387 for missingness (probability of observed data ≈0.99613 ), and $\Phi (-14.400)\approx 3.8\times 10^{-47}$ (probability of observed data ≈1 ).

In the following, the two significant sensitivity parameter estimates in the MNAR-cloglog reported in Table 1 are interpreted. The fixed effect of within-level missingness in the predictor on the outcome ( ${\hat{\mu }}_{ry.\phi }=-15.080$ , CrI = [ −17.430,−12.620 ]) indicates a strong negative association. Specifically, this estimate suggests that, after accounting for other modeled predictors, greater missingness in the daily interpersonal stress predictor is systematically associated with increased risk of suicidal behavior. That is, individuals with disproportionately high levels of missingness in their daily interpersonal stress data are more likely to report suicidal behavior, potentially reflecting avoidance, or disengagement on high-risk days. Substantial individual-level variability was observed in the effect, indicating potential heterogeneity in the missingness mechanisms ( ${\hat{\sigma }}_{ry.\phi }=3.642$ ). Furthermore, the fixed effect of within-level suicidal behavior on missingness in the outcome ( ${\hat{\mu }}_{y.\phi }=8.031$ , CrI = [ 7.181,9.502 ]) suggests that suicidal behavior tends to lead to increased missingness in suicidal behavior assessments, potentially due to participant withdrawal, avoidance, or distress. Moderate small variability across individuals in this effect was found ( ${\hat{\sigma }}_{y.\phi }=0.359$ ).

#### 4.3.2 Comparisons between MNAR-cloglog and MAR-cloglog

Comparing the MNAR-cloglog and MAR-cloglog substantive model for the outcome (suicidal behavior) in Table 1, the primary within-person daily interpersonal stress effect was significant in both but differs in magnitude: MNAR =1.114 (CrI =[0.309,1.940] ) versus MAR =1.754 (CrI =[0.760,2.812] ), that is, the MAR point estimate is about 1.58×(≈58%) larger on the cloglog scale, implying a stronger association when missingness is assumed at random. The peer victimization predictor was also significant in both models but with a smaller estimate under MAR— 0.770 (CrI =[0.008,1.780] ) versus MNAR 1.724 (CrI =[0.419,3.015] )—roughly 55% lower in magnitude. The linear survey-day trend was significant only under MNAR ( 0.007 , CrI $=[1.107\times 10^{-4},0.016]$ ; MAR: 0.004 , CrI =[−0.013,0.024] ), indicating some sensitivity to the missingness mechanism. All other fixed effects remain non-significant across the two models, while the intercept is significant only under MAR ( −7.036 , CrI =[−8.726,−5.659] ).

The MNAR–MAR differences are pronounced when comparing the MNAR-cloglog and MAR-cloglog missingness models for the outcome (suicidal behavior), as shown in Table 1. The intercept flipped sign and was significant in both models (MNAR: −1.701 , CrI =[−2.962,−0.538] ; MAR: 1.706 , CrI =[1.474,1.909] ), indicating very different baselines on the latent scale. Several predictors changed significance across missingness assumptions: the parental support predictor was significant only under MNAR ( 0.329 , CrI =[0.039,0.646] ; MAR: 0.001 , CrI =[−0.058,0.059] ), whereas the trauma history predictor was significant only under MAR ( 0.059 , CrI =[0.004,0.117] ; MNAR: 0.139 , CrI =[−0.133,0.441] ). The summer indicators were non-significant in MNAR (within: −0.487 , CrI =[−2.820,1.460] ; between: −0.038 , CrI =[−2.650,2.341] ) but strongly negative in MAR (within: −2.680 , CrI =[−3.279,−2.203] ; between: −2.205 , CrI =[−2.915,−1.569] ). The weekend effects were robustly negative in both models (within—MNAR: −3.372 , CrI =[−4.559,−2.356] ; MAR: −2.975 , CrI =[−3.455,−2.598] ; between—MNAR: −4.574 , CrI =[−7.303,−1.849] ; MAR: −13.600 , CrI =[−14.450,−12.810] ). Other predictors (suicidal ideation severity, linear/quadratic) remained non-significant in both models.

In the predictor model, results under the MNAR and MAR scenarios were generally comparable, as most parameter estimates and CrIs of fixed effects were similar across the two missing data assumptions (see Table 2).

#### 4.3.3 Comparisons between MNAR-cloglog and MNAR-logit

The statistical significance of predictors was compared across the MNAR-cloglog and MNAR-logit models using their respective 95% CrIs. However, the magnitudes of the regression coefficients are not directly comparable because each link function is defined on a different latent scale: the logit link models changes in log-odds, whereas the cloglog link models changes in the log cumulative hazard. These differing scales have distinct units and symmetry properties, such that identical coefficient values would imply different changes in the underlying event probability. Accordingly, the cross-model comparison focuses on the presence or absence of statistically significant associations rather than on direct magnitude comparisons.

Overall, results from the MNAR-cloglog and MNAR-logit models were highly concordant, with statistical-significance decisions overlapping for nearly all predictors. The few exceptions were limited to the following: (a) in the outcome model (Table 1), the survey-day effect was significant under MNAR-cloglog but not under MNAR-logit and (b) in the predictor model (Table 2), the sex predictor was significant under MNAR-logit but not under MNAR-cloglog. No differences in significance were observed in the corresponding missingness submodels. Taken together, these results indicate that, in this application, the choice of link function yields broadly similar conclusions, with only isolated predictors showing sensitivity to the link; this is consistent with prior findings (e.g., McCullagh & Nelder, 1989). Although the MNAR-cloglog and MNAR-logit models yielded similar patterns in the present dataset, the cloglog link was selected because it is theoretically consistent with hazard modeling, accommodates the asymmetric response pattern expected for rare events, and facilitates interpretation of coefficients as hazard ratios.

## 5 Simulation study

The aims of the simulation study are twofold in using the specified Bayesian selection model: (a) to evaluate parameter recovery of the model under conditions similar to our empirical study and (b) to assess the impact of treating missingness in the time-varying outcome and predictor as MAR on parameter recovery.

### 5.1 Simulation design and analysis

The data-generating model is given by the fitted model for the empirical study, with the estimates of MNAR-cloglog from Tables 1 and 2 considered as the true parameters under the condition of 106 persons in 90-day daily diary data. Patterns of missingness were generated according to four sensitivity parameters. In data generation, all predictors from the empirical study were held constant, with the exception of the daily interpersonal stress variables, which were generated from a predictor model. Five hundred datasets were generated.

For aim (a), the specified Bayesian selection model (MNAR-cloglog) was fitted to the generated datasets. For aim (b), the same model with the four sensitivity parameters ( $[\phi_{y.j},\;\phi_{x.j},\;\phi_{xy.j},\;\phi_{ry.j}{]}^{\prime }$ ) set to 0 (MAR-cloglog) was fitted to the same datasets. To evaluate the accuracy of the estimates, bias^3^ and root mean square error (RMSE) were calculated.

The same prior and hyperprior distributions used in the empirical study were used in the simulation study, with Bayesian estimation conducted in WinBUGS. No convergence problems were observed in any replication for either the MNAR-cloglog or MAR-cloglog models. To assess convergence, 10% of the 500 replications were examined. Based on the Gelman–Rubin statistic, with three chains using dispersed initial values, a burn-in of 5,000 iterations was applied, followed by 8,000 post-burn-in iterations for both the MNAR-cloglog and MAR-cloglog models. For all parameter estimates, the MCSE was smaller than 1% of the standard deviation of the estimates.

### 5.2 Simulation results

As shown in Table 3, parameter recovery for aim (a) was generally good under the MNAR-cloglog model. Overall, the MNAR-cloglog specification provided reliable recovery of most parameters, with bias and RMSE typically small to moderate, and the few large biases generally associated with coefficients whose true values were also large. For the daily interpersonal stress effects on the outcome ( $\gamma_{y.1}$ and $\gamma_{y.2}$ ) of interest, bias/RMSE for ${\hat{\gamma }}_{y.1}$ were 0.009/0.207 , and for ${\hat{\gamma }}_{y.2}$ were 0.053/0.217 , indicating negligible bias and acceptable precision. Across the remaining outcome coefficients in the outcome and outcome-missingness models under MNAR-cloglog, bias ranged from −0.845 to 1.320 and RMSE from 0.002 to 1.695 . Although some parameters displayed relatively large bias (e.g., $\mu_{ry.\phi }$ with bias 1.320 ), such values occurred for coefficients with moderate or large true effects, where the proportional impact is less concerning. Table 4 shows a similar pattern for the predictor and predictor-missingness models. Parameters in the predictor model were generally well recovered, with bias ranging from −0.241 to 0.406 and RMSE from 0.078 to 0.464 . For the predictor-missingness model, bias ranged from 0.001 to 0.217 and RMSE from 0.037 to 0.857 .

**Table 3:** Simulation study: Results of the outcome Table 3 long description.

	True		MNAR-cloglog			MAR-cloglog	
**Outcome**			Bias	RMSE		Bias	RMSE
*Substantive model*							
$\gamma_{y.0}$	−0.898		0.035	0.690		−3.325	3.509
$\gamma_{y.1}$	0.374		0.009	0.207		−0.220	0.350
$\gamma_{y.2}$	1.114		0.053	0.217		0.595	0.710
$\gamma_{y.3}$	−0.249		−0.054	0.402		0.378	1.006
$\gamma_{y.4}$	0.760		0.234	0.645		0.238	1.223
$\gamma_{y.5}$	−0.180		0.050	0.201		0.257	0.609
$\gamma_{y.6}$	−0.973		0.290	0.760		−2.503	2.913
$\gamma_{y.7}$	0.210		−0.092	0.160		0.602	1.569
$\gamma_{y.8}$	−1.308		0.010	0.980		0.014	1.117
$\gamma_{y.9}$	0.840		0.291	0.372		−0.431	0.780
$\gamma_{y.10}$	1.724		0.346	0.647		0.795	1.094
$\gamma_{y.11}$	−0.843		−0.204	0.529		−0.586	1.007
$\gamma_{y.12}$	0.150		−0.058	0.019		−0.778	1.040
$\gamma_{y.13}$	−0.405		0.101	0.349		0.383	0.900
$\gamma_{y.14}$	0.007		0.000	0.002		0.006	0.006
$\gamma_{y.15}$	−0.176		0.012	0.128		−3.363	3.544
$\gamma_{y.16}$	−0.107		0.210	0.378		1.277	1.452
SD of $u_{y.0}$	4.069		−0.753	0.936		0.854	1.103
$\mu_{ry.\phi }$	−15.080		1.320	1.443		-	-
$\sigma_{ry.\phi }$	3.642		−0.122	0.675		-	-
*Missing model*							
$\omega_{y.0}$	−1.701		−0.550	0.766		1.055	1.078
$\omega_{y.1}$	−0.265		−0.011	0.173		0.262	0.271
$\omega_{y.2}$	0.139		−0.059	0.184		−0.204	0.213
$\omega_{y.3}$	0.329		−0.098	0.199		−0.234	0.240
$\omega_{y.4}$	0.140		−0.132	0.436		0.160	0.233
$\omega_{y.5}$	−0.487		0.073	0.194		−0.202	0.220
$\omega_{y.6}$	−0.038		0.010	0.430		0.014	0.446
$\omega_{y.7}$	−3.372		0.063	0.368		1.762	1.764
$\omega_{y.8}$	−4.574		−0.829	1.695		1.760	1.891
$\omega_{y.9}$	0.142		−0.065	0.190		−0.125	0.138
SD of $a_{y.j}$	0.435		−0.094	0.153		0.164	0.170
$\mu_{y.\phi }$	8.031		−0.845	1.091		-	-
$\mu_{xy.\phi }$	−0.663		0.182	0.302		-	-
$\sigma_{y.\phi }$	0.359		0.037	0.320		-	-
$\sigma_{xy.\phi }$	0.497		0.153	0.386		-	-

*Note*: - indicates that the effect was not modeled.

**Table 4:** Simulation study: Results of the predictor Table 4 long description.

**Predictor**	True		MNAR-cloglog			MAR-cloglog	
			Bias	RMSE		Bias	RMSE
*Substantive model*							
$\gamma_{x.0}$	−1.339		−0.241	0.318		−0.101	0.193
$\gamma_{x.1}$	−0.022		−0.018	0.095		0.014	0.110
$\gamma_{x.2}$	0.009		−0.044	0.100		−0.073	0.111
$\gamma_{x.3}$	0.356		0.099	0.227		−0.013	0.209
$\gamma_{x.4}$	−0.308		−0.138	0.270		−0.035	0.243
$\gamma_{x.5}$	0.273		0.406	0.464		0.417	0.465
$\gamma_{x.6}$	−0.134		−0.025	0.102		−0.035	0.104
$\gamma_{x.7}$	0.278		−0.039	0.183		−0.045	0.176
$\gamma_{x.8}$	0.254		−0.027	0.139		−0.066	0.144
$\gamma_{x.9}$	0.013		0.009	0.130		0.062	0.161
$\gamma_{x.10}$	0.116		0.024	0.114		−0.002	0.139
$\gamma_{x.11}$	−0.068		0.060	0.106		0.062	0.101
$\gamma_{x.12}$	0.054		−0.179	0.202		−0.211	0.232
$\gamma_{x.13}$	−0.087		−0.060	0.153		−0.045	0.130
$\gamma_{x.14}$	−0.077		0.070	0.137		0.057	0.129
SD of $u_{x.0}$	0.782		0.005	0.078		0.006	0.075
*Missing model*							
$\omega_{x.0}$	1.851		0.006	0.106		−0.081	0.005
$\omega_{x.1}$	0.054		0.001	0.037		0.005	0.001
$\omega_{x.2}$	−3.127		0.109	0.532		0.461	0.094
$\omega_{x.3}$	−2.662		0.019	0.415		0.030	0.160
$\omega_{x.4}$	−3.232		0.034	0.456		0.443	0.030
$\omega_{x.5}$	−14.400		0.217	0.857		0.820	0.190
SD of $a_{x.j}$	0.223		0.001	0.044		0.001	0.040
$\mu_{x.\phi }$	−0.150		0.015	0.156		-	-
$\sigma_{x.\phi }$	0.522		0.032	0.232		-	-

*Note*: - indicates that the effect was not modeled.

Comparisons between the MNAR-cloglog and MAR-cloglog models for aim (b) highlight the consequences of assuming MAR. In the outcome and outcome-missingness models (Table 3), ignoring sensitivity parameters in MAR-cloglog led to greater bias and RMSE, including for the daily interpersonal stress effects ( −0.220/0.350 for ${\hat{\gamma }}_{y.1}$ and 0.595/0.710 for ${\hat{\gamma }}_{y.2}$ ), relative to MNAR-cloglog. In the predictor-missingness model (Table 4), MAR-cloglog produced larger bias in the fixed-effect estimates than MNAR-cloglog, although adding sensitivity parameters in MNAR-cloglog increased RMSE compared to MAR-cloglog.

## 6 Summary and discussion

This study specified a Bayesian selection model tailored to the analysis of binary time-series daily diary data, characterized by rare outcomes and nonmonotone, nonignorable missingness in both the time-varying outcome and predictor. The Bayesian framework, implemented via WinBUGS, enabled joint estimation of model parameters and imputation of missing data while accounting for uncertainty in both the missing data mechanism and model structure. The approach was applied to a motivating empirical dataset to examine the relationship between daily interpersonal stress and suicidal behavior. In addition, a simulation study demonstrated the recovery of parameters and highlighted biased estimates of focal parameters that arose from ignoring sensitivity parameters in the outcome model and its associated missingness model. Together, the empirical and simulation results support the applicability of the specified model for addressing methodological challenges in longitudinal studies involving complex missing data structures.

### 6.1 What did we learn from the specified models?

The present findings provide insights into the challenges and complexities of investigating suicidal behavior using intensive longitudinal designs, with substantive implications for understanding the timing of effects of stress and peer victimization on suicidal behavior risk. These findings primarily demonstrate how the proposed model captures both within-person dynamics and between-person differences in this empirical study.

The results from the Bayesian selection models suggest that within-person daily interpersonal stress fluctuations are associated with suicidal behavior. Specifically, a one-unit increase in daily interpersonal stressor exposure was associated with a 205% increase in the hazard of suicidal behavior. In contrast, the effect of prior-day stress was not statistically significant. This pattern highlights the potential importance of acute, immediate interpersonal stress while suggesting a weaker or absent lagged effect. Furthermore, the results illustrate the interplay between stable, between-person risk factors and dynamic, within-person processes. Consistent with prior research, exposure to overt peer victimization (reported at baseline) was associated with a 461% increase in the hazard of suicidal behavior for a one-standard-deviation increase in peer victimization.

These results also provide insights into factors that predict missingness in intensive longitudinal designs with high-risk clinical samples. Based on the coding of reasons for missingness, study dropout and the need for higher levels of care appear to be key contributors to missing data. In addition, participants commonly endorsed forgetting to complete the survey, despite text message reminders, as well as engagement in competing activities. Quantitatively, parental support, weekend days, and summer months were associated with lower levels of missingness, highlighting factors that may help strengthen completion rates.

### 6.2 Discussion and methodological limitations of the current study

In this study, a parametric substantive model is chosen for the selection model because the parametric effects of relationships between daily interpersonal stress and suicidal behavior are the primary parameters of interest, and a missing data model is considered to account for the dependence between the outcome and missing data processes. In this parametric selection model, different assumptions on the $\phi_{j}$ (the effect of $y_{tj}$ on the latent response $r_{y.tj}^{*}$ ) yield different results because the likelihood is a function of the $\phi_{j}$ . Little and Rubin (2020) suggested fixing the $\phi_{j}$ at reasonable values, potentially informed by expert opinion, and then maximizing the observed data log-likelihood over the remaining parameters. Alternatively, in a frequentist analysis, the maximum likelihood estimate of $\phi_{j}$ can be used as the best fit to the observed data within a given parametric specification. In Bayesian analysis, different prior distributions for sensitivity parameters can be considered as part of a sensitivity analysis. However, strong prior knowledge about sensitivity parameters was not available in the current study. Therefore, weakly informative priors are adopted to reflect uncertainty without unduly influencing the posterior distribution. This approach balances prior plausibility and empirical learning by regularizing extreme parameter estimates that may arise from sparse or highly missing data, while allowing the data to inform the posterior inference to the extent possible. This strategy is consistent with recommendations for sensitivity parameter estimation in nonignorable missing data models when expert elicitation is difficult or unreliable (e.g., Daniels & Hogan, 2008). Other specifications of sensitivity parameters could be considered as part of future work, particularly when cumulative empirical evidence or external validation studies become available. In such cases, Bayesian analysis provides a principled framework for incorporating increasingly informative priors, allowing researchers to formally integrate substantive knowledge into the estimation process while maintaining coherence with the observed data.

In the present study, model specification is primarily motivated by the predominance of MNAR processes, as evidenced by our systematic coding of potential reasons for missingness, though our systematic coding also indicates that some missingness may be consistent with MAR. As noted by Gomer and Yuan (2023), it is likely that a mixture of missing-data mechanisms underlies missing values in a dataset. To address this complexity, a hierarchical Bayesian model incorporates a person-specific coefficient that quantifies the effect of data on the latent response governing missingness. It should be noted, however, that a limitation of the current approach is the assumption that this coefficient is constant over time; when additional data become available, extending the model to incorporate a time-varying coefficient would be an interesting direction for future research.

Missing models for MNAR, including a Bayesian selection model presented in the current study, are highly dependent on distributional assumptions or model specifications (Little, 1995; Kenward, 1998). Thus, content knowledge is crucial for exploring the missing data mechanism. The methodological development of the current study was guided by close collaboration with clinical psychology experts in adolescent suicide risk and informed by a thorough review of current literature and clinical experience in suicide research. First, during data collection, the primary reason for missingness was systematically coded, revealing that most cases were assumed to be MNAR. Second, predictor selection for both substantive and missing data models was guided by theoretical relevance and statistical considerations. As presented in Appendix C, predictors were chosen based on content knowledge, ensuring alignment with established risk factors for suicidal behavior and missingness patterns, as well as daily interpersonal stress levels and their associated missingness patterns. In addition to these theoretical considerations, a statistical recommendation is also incorporated by including auxiliary variables (predictors related to missingness but not directly to the outcome) to improve model robustness, reduce bias, and better differentiate MNAR from MAR mechanisms, thereby strengthening the validity of sensitivity analyses (e.g., Little & Rubin, 2020). In addition, when an additional model—such as a predictor model—is considered as an extension of a traditional selection model (which typically focuses only on outcomes), a continuous predictor was included in the missingness model for outcomes, even if it does not appear in the predictor model, as a strategy for identification (Chen et al., 2018).

In the current study, relevant confounders affecting the observed data (suicidal behavior and stress) and their missingness are assumed to be adequately accounted for, based on a thorough review of current literature and clinical experience in suicide research. Furthermore, adolescents without suicidal behavior across the 90-day survey period were selected based on the same criteria as those who later endorse suicidal behavior. As a result, inferences from this study are valid within a clinical population—namely, adolescents followed after acute psychiatric treatment for NSSI, suicidal ideation, and/or suicidal behaviors.

Therefore, given the control of observed confounders, we assume residual independence between the suicidal behavior model and its missingness model, as well as between the interpersonal stress model and its missingness model. Chen et al. (2018) demonstrated through theoretical derivations and a simulation study that the estimates of regression coefficients in the outcome model (i.e., focal parameters) are robust to misspecification of the joint error distributions in the extended selection model, which assumes a multivariate normal distribution. Because the error distribution in the outcome model of the current study follows a Gumbel distribution, the correlation between the errors in the outcome and missing model is more complicated; however, it can be calculated as $\rho =-\frac{\sqrt{6}}{\pi }\int_{0}^{1}\ln[-\ln(u)]\;\Phi^{-1}(u)\;du$ (where u∼Uniform(0,1) . Future research on the misspecification of error distributions is needed in our specified Bayesian selection model.

Because the current study is a case study, the simulation study was designed as the primary basis for evaluating the feasibility, parameter recovery, and complexity of the proposed model under the same conditions as the empirical application. The applied example is therefore intended to serve as an illustration of how the model can be implemented in practice, rather than as a basis for confirmatory inference. Accordingly, the findings should be interpreted with caution, as they may not generalize beyond the examined scenarios. To establish broader applicability, additional simulation studies are needed to assess how the model performs under different conditions, such as varying predictor effects, different sample sizes, alternative distributions of errors, or different patterns of missing data. In particular, as sample sizes increase, issues associated with analyzing rare binary outcomes tend to diminish (Allison, 2012). Expanding the scope of simulations would help ensure the robustness and generalizability of the findings across a wider range of research contexts.

### 6.3 Conclusion and broad impact of the current study

Despite methodological limitations, the present study contributes to the methodological literature by developing a Bayesian selection modeling framework for analyzing binary time-series data with rare outcomes and nonmonotone, nonignorable missingness. This work has broad implications for psychological, behavioral, and health sciences, where daily diary methods are increasingly used to study low-frequency but clinically meaningful events, such as suicidal behavior. Ignoring the non-ignorable nature of missingness can lead to biased estimates and diminished validity of scientific conclusions. The proposed modeling framework offers a principled solution that improves the robustness and interpretability of estimates under these challenging conditions. Future research can extend this framework to multivariate settings, incorporate additional sources of time-varying or hierarchical structure. Overall, this study advances the methodological toolkit available to researchers working with complex longitudinal data and supports the development of more valid and nuanced scientific understanding of dynamic processes in daily life.

## Figures and Tables

**Appendix B:**
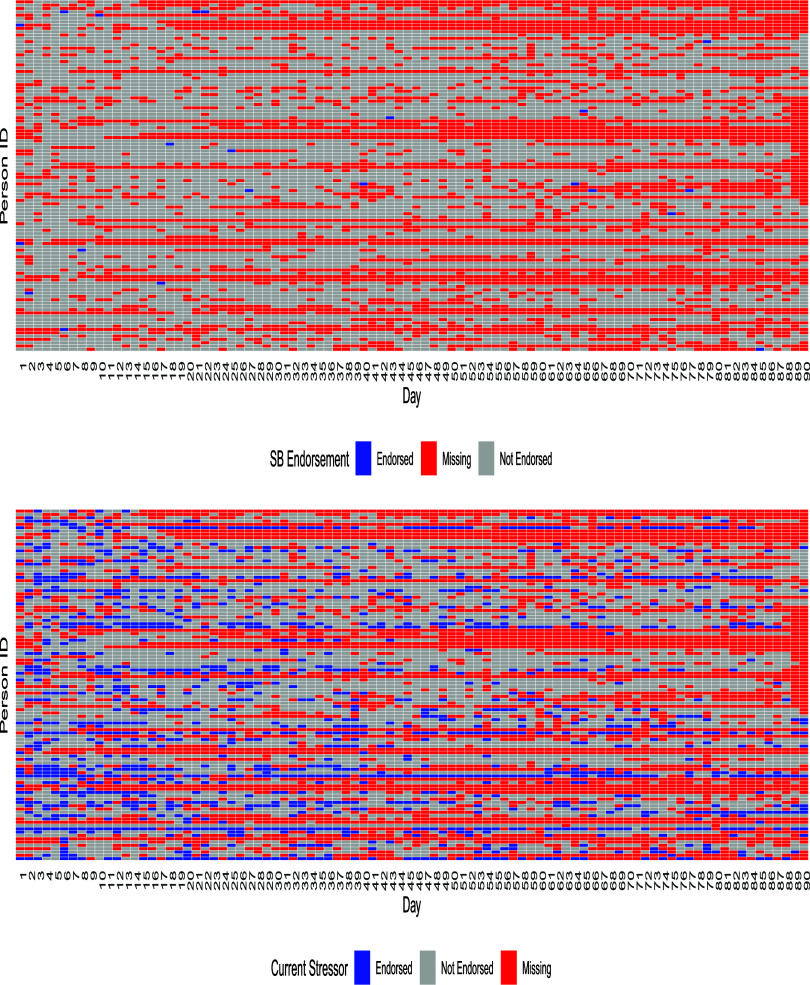
Missing data patterns Note: Suicidal behaviors (top) and the current daily interpersonal stress variable (bottom). Appendix B: long description.

**Appendix D:**
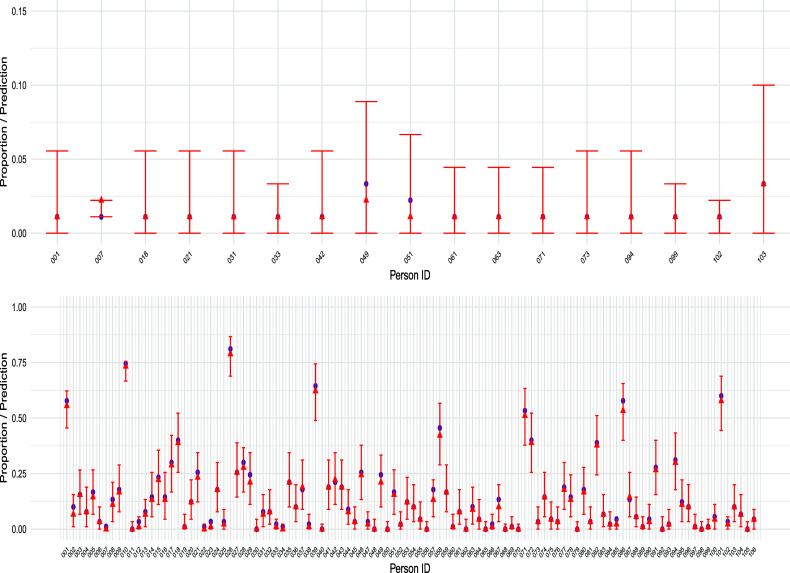
Posterior predictive checking of a selected model for observed data ( $[y_{j}^{o},x_{j}^{o}{]}^{\prime }$ ) Note: In both panels, blue dots represent observed person-level proportions, red triangles indicate posterior median predictions, and red error bars denote 95% credible intervals (CrIs). Appendix D: long description.

## Data Availability

The WinBUGS code used in the illustration is available on the Open Science Framework at https://osf.io/4ctqj. The data used in the illustration are available through the National Institute of Mental Health (NIMH) Data Archive at 10.15154/50rv-qk23.

## Footnotes

1 The authors in the current study also considered rstan (Stan Development Team, 2024), which implements sampling algorithms designed for efficient exploration of high-dimensional posterior distributions. However, we used WinBUGS for data augmentation in sampling because rstan does not allow discrete unknowns in the parameters block (Stan Development Team, 2025); therefore, discrete missing outcomes and predictors ( $y_{j}^{m}$ and $x_{j}^{m}$ ) cannot be sampled directly and must be marginalized explicitly from the likelihood (i.e., coded as a sum over their support in the model block).

2 When MNAR-probit models were considered, there were “traps” in WinBUGS, suggesting poor identification or numerical instability of the MNAR-probit specification under our data and priors. A potential reason for this is that the narrower latent scale of the probit model, with a fixed variance of 1, makes it more susceptible to identifiability issues in complex models, whereas the logit model’s larger variance (3.29) can be more forgiving for the sampler.

3 Relative bias was not reported. The measure is ill-behaved when small denominators yield spuriously large percentages, which can mischaracterize estimator performance. Accordingly, evaluation focused on bias and RMSE.

## Appendix

**Appendix A:** Descriptive statistics of variables Appendix A: long description.

	Mean(SD)	Frequency(%)
**Outcome**		
Suicidal behavior (time varying)		
- 0 (No)		5821(61.02)
- 1 (Yes)		22(0.23)
- NA		3697(38.75)
**Predictor**		
*Focal (time varying)*		
Daily interpersonal stress		
- 0 (No)		4506(47.24)
- 1 (Yes)		1336(14.00)
- NA		3698(38.76)
*Demographic factors (time invariant)*		
Age in months[9.43]	187(16.1)	
Sex		
- 0 (Male)		33(31.13)
- 1 (Female)		73(68.87)
Gender minority		
- 0 (Not endorsed)		80(75.47)
- 1 (Endorsed)		21(19.81)
- NA		5(4.72)
Sexual minority		
- 0 (Not endorsed)		50(47.17)
- 1 (Endorsed)		51(48.11)
- NA		5(4.72)
*Baseline (time invariant)*		
Suicidal ideation severity[5.7]	35.3(22.5)	
Number of past suicide attempts[11.3]	1.83(3.11)	
Trauma history[12.3]	2.52(1.81)	
Peer victimization		
- Overt peer victimization[17]	4.85(2.5)	
- Relational peer victimization[17]	6.78(3.37)	
- Reputational peer victimization[17]	7.78(4.28)	
Support		
- Parental support[10.4]	41.6(13.4)	
- Peer/friend support[10.4]	51.1(13.0)	
Depression[10.4]	24.3(7.39)	
Anxiety[10.4]	29.0(9.09)	
Coping flexibility		
- Multiple coping strategy use[11.3]	22.1(9.45)	
- Situational coping[11.3]	29.2(8.66)	
- Coping rigidity[11.3]	22.8(8.66)	
Sleep		
- Sleep disturbance[6.6]	64.5(7.2)	
- Sleep impairment[6.6]	25.2(7.59)	
Non-suicidal self injury (NSSI)		
- No		16(15.09)
- Yes		77(72.65)
- NA		13(12.26)
*Study characteristics (time varying)*		
Time: Day		45.5(25.98)
Time: Summer		
- 0 (No)		8922(93.522)
- 1 (Yes)		618(6.478)
Time: Weekends		
- 0 (No)		7868(82.474)
- 1 (Yes)		1672(17.526)

*Note*: NA indicates missingness for categorical variables; values in brackets indicate the percentage of missingness for continuous variables.

## Appendix

**Appendix C:** Predictor ( w ) selection Appendix C: long description.

Predictor	Outcome model		Predictor model		Missing model for outcome		Missing model for predictor
Baseline suicidal ideation severity	✓				✓		✓
	Liu et al. (2022)				Jacobucci et al. (2024)		Jacobucci et al. (2024)
Baseline number of	✓						
past suicide attempts	Cantor et al. (2023)						
Demographic factors	✓		✓				
(Age, sex/gender minority status)	Cantor et al. (2023)		Liu et al. (2022)				
	Miranda-Mendizabal et al. (2019)		Santee et al. (2023)				
Baseline trauma history	✓				✓		
	Grummitt et al. (2024); Liu et al. (2022)				Levi-Belz et al. (2018)		
Baseline	✓						
Peer victimization	Giacomo et al. (2018)						
	John et al. (2018)						
	Richardson et al. (2024)						
Baseline parental support	✓		✓		✓		
	Miller et al. (2015)		Mackin et al. (2017)		Levi-Belz et al. (2018)		
	Pérez-Albéniz et al. (2023)						
Baseline depression & anxiety			✓				
			Liu (2013)				
			Liu and Alloy (2010)				
			Liu et al. (2024)				
Baseline coping flexibility			✓				
			Santee et al. (2023)				
Baseline peer/friend support			✓				
			Mackin et al. (2017)				
Baseline sleep			✓				
			Slavish et al. (2021)				
Time:					✓		✓
Days receiving daily surveys					Jacobucci et al. (2024)		Jacobucci et al. (2024)
Non-suicidal self injury					✓		✓
					Bloom et al. (2024)		Bloom et al. (2024)
Time: Summer					✓		✓
					Bloom et al. (2024)		Bloom et al. (2024)
Time: Weekends					✓		✓
					Bloom et al. (2024)		Bloom et al. (2024)

*Note*: ✓ indicates that the predictor was included in the corresponding model.

## Table 1 Long description

Beginning with the substantive model, rows list outcomes including intercept, prior stress within, current stress within, suicidal ideation severity, number of past suicide attempts, quadratic of past suicide attempts, interview age, quadratic of interview age, sex, gender minority, sexual minority, trauma history, peer victimization, relational peer victimization, reputational relational peer victimization, parental support, survey day, standard deviation of intercept, mean and standard deviation of MNAR effect for x and y. For each outcome, columns display median and credible interval values for MNAR-cloglog, MAR-cloglog, and MNAR-logit models. For example, the intercept under MNAR-cloglog shows a median of 0.898 and credible interval of negative 2.635 to 0.789; MAR-cloglog median is negative 7.036 with credible interval negative 8.726 to negative 5.659; MNAR-logit median is negative 0.109 with credible interval negative 2.110 to 2.150. Significant effects are bolded, as noted in the table footnote. The missing model section follows, listing outcomes such as intercept, suicidal ideation severity, quadratic of suicidal ideation severity, trauma history, parental support, N S S I, summer within and between, weekend within and between, standard deviation of random intercept, mean and standard deviation of MNAR effect for x and y. Each outcome is presented with corresponding median and credible interval values for MNAR-cloglog, MAR-cloglog, and MNAR-logit models, with dashes indicating effects not modeled. The table footnote clarifies that bold indicates significance based on the 95 percent credible interval and dash denotes effects not modeled.

Navigate back to Table 1.

## Table 2 Long description

Beginning at the top, the table is divided into two main sections: substantive model and missing model. Each section lists predictors in the leftmost column, including intercept, suicidal ideation severity, interview age, sex, gender minority, sexual minority, parental support, depression symptoms, anxiety symptoms, coping flexibility (multiple coping, situational coping, coping rigidity), peer or friend support, sleep disturbance, sleep impairment, and random intercept standard deviation. For each predictor, three sets of columns are provided: MNAR-cloglog, MAR-cloglog, and MNAR-logit. Each set contains median and credible interval (CrI) values. Significant effects are indicated in bold. For example, in the substantive model, the intercept median for MNAR-cloglog is minus one point three three nine with CrI from minus one point six two three to minus zero point nine five five. Suicidal ideation severity has a median of zero point zero two two and CrI from minus zero point two six zero to zero point two one three. The missing model section follows, listing predictors such as intercept, suicidal ideation severity, summer within, summer between, weekend within, weekend between, and random intercept standard deviation, with corresponding median and CrI values. Effects not modeled are marked with a dash. The table footnote clarifies that bold indicates significance based on the ninety-five percent credible interval and dash indicates the effect was not modeled.

Navigate back to Table 2.

## Table 3 Long description

The table is organized with outcome parameters as rows and model metrics as columns. The first section, labeled substantive model, lists gamma sub y dot 0 to gamma sub y dot 16. For each, the columns are: True value, Bias and R M S E for MNAR-cloglog, and Bias and R M S E for MAR-cloglog. For example, gamma sub y dot 0 has True 0.898, MNAR-cloglog Bias 0.035 and R M S E 0.690, MAR-cloglog Bias -3.325 and R M S E 3.509. The pattern continues for all gamma parameters. Additional rows include S D of u sub y dot 0 (True 4.069, MNAR-cloglog Bias -0.753, R M S E 0.936, MAR-cloglog Bias 0.854, R M S E 1.103), mu sub r y dot phi (True -15.080, MNAR-cloglog Bias 1.320, R M S E 1.443), sigma sub r y dot phi (True 3.642, MNAR-cloglog Bias -0.122, R M S E 0.675). The next section, labeled missing model, lists omega sub y dot 0 to omega sub y dot 9, each with True, Bias and R M S E for both models. For example, omega sub y dot 0 has True -1.701, MNAR-cloglog Bias -0.550, R M S E 0.766, MAR-cloglog Bias 1.055, R M S E 1.078. Additional rows include S D of a sub y dot j (True 0.435, MNAR-cloglog Bias -0.094, R M S E 0.153, MAR-cloglog Bias 0.164, R M S E 0.170), mu sub y dot phi (True 8.031, MNAR-cloglog Bias -0.845, R M S E 1.091), mu sub x y dot phi (True -0.663, MNAR-cloglog Bias 0.182, R M S E 0.302), sigma sub y dot phi (True 0.359, MNAR-cloglog Bias 0.037, R M S E 0.320), sigma sub x y dot phi (True 0.497, MNAR-cloglog Bias 0.153, R M S E 0.386). Dashes indicate effects not modeled. All values are reported to three decimal places.

Navigate back to Table 3.

## Table 4 Long description

The table is structured with predictors listed vertically and model conditions horizontally. The first section, substantive model, includes predictors gamma sub x dot 0 through gamma sub x dot 14. For each, columns show true values, bias, and R M S E under M N A R-cloglog and M A R-cloglog. For example, gamma sub x dot 0 has true value 0.339, bias -0.241 and R M S E 0.318 for M N A R-cloglog, bias -0.101 and R M S E 0.193 for M A R-cloglog. The missing model section lists omega sub x dot 0 through omega sub x dot 5, with similar columns. For instance, omega sub x dot 0 has true value 1.851, bias 0.006 and R M S E 0.106 for M N A R-cloglog, bias -0.081 and R M S E 0.005 for M A R-cloglog. Standard deviations for u sub x dot 0 and a sub x dot j are also provided. The final rows show mu sub x dot phi and sigma sub x dot phi with values and bias/R M S E, where dashes indicate effects not modeled. The note clarifies that '-' means the effect was not modeled.

Navigate back to Table 4.

## Appendix A Long description

Beginning with outcomes, suicidal behavior is reported as 5821 cases with no behavior (61.02 percent), 22 cases with behavior (0.23 percent), and 3697 missing (38.75 percent). Predictors include daily interpersonal stress, with 4506 reporting no stress (47.24 percent), 1336 reporting stress (14 percent), and 3698 missing (38.76 percent). Demographic factors show age in months with a mean of 187 and standard deviation of 16.1. Sex is divided into 33 males (31.13 percent) and 73 females (68.87 percent). Gender minority status is not endorsed by 80 (75.47 percent), endorsed by 21 (19.81 percent), and missing for 5 (4.72 percent). Sexual minority status is not endorsed by 50 (47.17 percent), endorsed by 51 (48.11 percent), and missing for 5 (4.72 percent). Baseline variables include suicidal ideation severity with a mean of 35.3 and standard deviation of 22.5, number of past suicide attempts with a mean of 1.83 and standard deviation of 3.11, and trauma history with a mean of 2.52 and standard deviation of 1.81. Peer victimization is subdivided into overt (mean 4.85, SD 2.5), relational (mean 6.78, SD 3.37), and reputational (mean 7.78, SD 4.28). Support is measured as parental support (mean 41.6, SD 13.4) and peer/friend support (mean 51.1, SD 13.0). Depression has a mean of 24.3 and SD of 7.39, anxiety has a mean of 29.0 and SD of 9.09. Coping flexibility includes multiple coping strategy use (mean 22.1, SD 9.45), situational coping (mean 29.2, SD 8.66), and coping rigidity (mean 22.8, SD 8.66). Sleep disturbance has a mean of 64.5 and SD of 7.2, sleep impairment has a mean of 25.2 and SD of 7.59. Non-suicidal self injury is reported as 16 cases with no injury (15.09 percent), 77 cases with injury (72.65 percent), and 13 missing (12.26 percent). Study characteristics include time as day (mean 45.5, SD 25.98), summer with 8922 not in summer (93.522 percent) and 618 in summer (6.478 percent), weekends with 7868 not on weekends (82.474 percent) and 1672 on weekends (17.526 percent). Missingness is indicated for categorical and continuous variables as noted.

Navigate back to Appendix A.

## Appendix B Long description

The top panel is a heatmap with Person I D on the y-axis and Day on the x-axis, showing S B Endorsement. Each cell represents a person-day, colored blue for endorsed, red for missing, and gray for not endorsed. Most cells are gray, with scattered blue and red, indicating rare endorsement and frequent missing data. The bottom panel is a similar heatmap for Current Stressor, with the same axes. Here, blue indicates endorsed, gray not endorsed, and red missing. Blue cells are more frequent and widely distributed compared to the top panel, suggesting higher endorsement rates for stressors. Legends below each panel clarify the color coding.

Navigate back to Appendix B.

## Appendix C Long description

The table consists of multiple columns: Predictor, Outcome model, Predictor model, Missing model for outcome, and Missing model for predictor. The leftmost column lists predictors such as baseline suicidal ideation severity, baseline number of past suicide attempts, demographic factors (age, sex/gender minority status), baseline trauma history, peer victimization, parental support, depression and anxiety, coping flexibility, peer/friend support, sleep, and time variables (days receiving daily surveys, summer, weekends, non-suicidal self injury). For each predictor, the corresponding columns display either a check mark or a citation indicating inclusion in the model. For example, baseline suicidal ideation severity is included in the outcome model (check mark), missing model for outcome (check mark), and missing model for predictor (check mark), with citations to Liu et al. (2022) and Jacobucci et al. (2024). Demographic factors are included in outcome and predictor models, with citations to Cantor et al. (2023), Liu et al. (2022), Miranda-Mendizabal et al. (2019), and Santee et al. (2023). Peer victimization is included in the outcome model with citations to Giacomo et al. (2018), John et al. (2018), and Richardson et al. (2024). Parental support is included in outcome, predictor, and missing models, with citations to Miller et al. (2015), Mackin et al. (2017), Levi-Belz et al. (2018), and Pérez-Albéniz et al. (2023). Depression and anxiety, coping flexibility, peer/friend support, and sleep are included in predictor models with citations to Liu (2013), Liu and Alloy (2010), Liu et al. (2024), Santee et al. (2023), Mackin et al. (2017), and Slavish et al. (2021). Time variables and non-suicidal self injury are included in missing models for outcome and predictor, with citations to Jacobucci et al. (2024) and Bloom et al. (2024). The note at the bottom clarifies that a check mark indicates inclusion of the predictor in the corresponding model.

Navigate back to Appendix C.

## Appendix D Long description

The top panel displays a line graph with Person I D values 001 to 103 along the x-axis and Proportion forward slash Prediction on the y-axis, ranging from 0.00 to 0.15. Each Person I D is marked with a blue dot for observed proportion, a red triangle for posterior median prediction, and a red vertical error bar representing the 95 percent credible interval. Most Person I D values cluster near zero, with wide credible intervals, and a few higher values at the right end. The bottom panel shows a similar structure but with Person I D values densely packed from 001 to 103 along the x-axis and the y-axis ranging from 0.00 to 1.00. Blue dots and red triangles are distributed more variably, with some Person I D values showing high observed and predicted proportions, and error bars of varying lengths. The overall trend in both panels is that observed and predicted values are generally close, but credible intervals are wide for many Person I D values, indicating uncertainty.

Navigate back to Appendix D.
